# Allogenic mitochondria transfer improves cardiac function in iPS-cell-differentiated cardiomyocytes of a patient with Barth syndrome

**DOI:** 10.1038/s12276-025-01472-7

**Published:** 2025-06-24

**Authors:** Ye Seul Kim, Sukdong Yoo, Yoon Ji Jung, Jung Won Yoon, Yong Seong Kwon, Nayeon Lee, Chong Kun Cheon, Jae Ho Kim

**Affiliations:** 1 Department of Physiology, School of Medicine, Pusan National University, Yangsan, Republic of Korea; 2 Division of Medical Genetics and Metabolism, Department of Pediatrics, School of Medicine, Pusan National University, Pusan National University Children’s Hospital, Yangsan, Republic of Korea; 3 Research Institute for Convergence of Biomedical Science and Technology, Pusan National University Yangsan Hospital, Yangsan, Republic of Korea

**Keywords:** Heart stem cells, Mitochondria, Disease model

## Abstract

Barth syndrome (BTHS) is an ultrarare, infantile-onset, X-linked recessive mitochondrial disorder that primarily affects males, owing to mutations in *TAFAZZIN*, which catalyzes the remodeling of cardiolipin, a mitochondrial phospholipid required for oxidative phosphorylation. Mitochondrial transplantation is a novel technique to treat mitochondrial dysfunction by delivering healthy mitochondria to diseased cells or tissues. Here we explored the possibility of using stem-cell-derived cardiomyocytes as a source of mitochondrial transplantation to treat BTHS. We established induced pluripotent stem (iPS) cells from healthy individuals and from patients with BTHS and differentiated them into cardiomyocytes. The iPS-cell-differentiated cardiomyocytes (CMs) derived from patients with BTHS exhibited less expression of cardiomyocytes markers, such as α-SA, cTnT and cTnI, and smaller cell size than normal iPS-cell-derived CMs. Multielectrode array analysis revealed that BTHS CMs exhibited shorter beat period and longer field potential duration than normal CMs. In addition, mitochondrial morphology and function were impaired and mitophagy was decreased in BTHS CMs compared with normal CMs. Transplantation of mitochondria isolated from normal CMs induced mitophagy in BTHS CMs, mitigated mitochondrial dysfunction and promoted mitochondrial biogenesis. Furthermore, mitochondrial transplantation stimulated cardiac maturation and alleviated cardiac arrhythmia of BTHS CMs. These results suggest that normal CMs are useful for allogeneic transplantation in the treatment of mitochondrial diseases, including BTHS.

## Stem-cell-derived mitochondria improve Barth syndrome cardiomyocytes

Barth syndrome is a rare genetic disorder that mainly affects boys, causing heart problems, muscle weakness and growth delays. The condition is linked to mutations in the TAFAZZIN gene, which disrupts normal mitochondrial function. In this study, scientists used induced pluripotent stem cells from both healthy individuals and patients with Barth syndrome. They differentiated these induced pluripotent stem cells into heart cells (cardiomyocytes) and then transferred mitochondria from healthy cells to those affected by Barth syndrome. This process aimed to restore mitochondrial function and improve cell health. The results showed that mitochondrial transfer improved mitochondrial function and reduced oxidative stress in affected cells. It also enhanced the structural and electrical properties of the heart cells, suggesting potential therapeutic benefits. The researchers concluded that mitochondrial transfer could be a promising approach for treating Barth-syndrome-related cardiac issues.

This summary was initially drafted using artificial intelligence, then revised and fact-checked by the author.

## Introduction

Barth syndrome (BTHS), reported in 1979^1^, is a rare X-linked genetic disease that primarily affects boys^2^. It is characterized by various symptoms, including cardiomyopathy, neutropenia, skeletal muscle weakness and growth delay^1,3^. Patients may also experience fatigue and exercise intolerance^4,5^. Treatment is mainly supportive and aims to manage symptoms such as heart failure^6–8^, with medications and lifestyle modifications. BTHS is caused by mutations in *TAFAZZIN* (*HGNC: l11577 [MIM 300394], TAZ*) gene, which is a protein associated with the inner mitochondrial membrane^9^. It is required for the normal biogenesis of cardiolipin, which is a signature mitochondrial phospholipid^10^. The characteristic fatty acid composition of cardiolipin promotes its association with proteins in the inner mitochondrial membrane, facilitating the formation of the mitochondrial electron transport chain system. Defects in cardiolipin metabolism are responsible for mitochondrial function deficiencies in BTHS^11^. Accumulation of damaged mitochondria in the cells of patients with BTHS leads to oxidative stress, which contributes to the development of cardiomyopathy^12,13^.

Mitophagy, a type of autophagy, removes and degrades damaged mitochondria and plays a key role in mitochondrial quality control^14,15^. During mitophagy, damaged mitochondria are enclosed by a double-membrane structure, the phagophore, which forms an autophagosome and fuses with the lysosome to target its contents for degradation^16^. Externalized cardiolipin on injured mitochondria recruits autophagic machinery to the mitochondria through interaction with LC3^17^. Cardiolipin deficiency in BTHS is also associated with impaired mitophagy^13,18–21^. *TAZ* knockdown causes defective mitophagy, leading to the accumulation of autophagic vacuoles and dysfunctional mitochondria in the hearts of *TAZ*-knockdown mice and mouse embryonic fibroblasts^22,23^. Furthermore, restoration of mitophagy by rapamycin treatment mitigated mitochondrial dysfunction and dilated cardiomyopathy^22^. Therefore, targeting mitophagy may represent a potential therapeutic strategy for treating BTHS-associated cardiac dysfunction^12,22,24,25^.

Mitochondrial transfer, the exchange of mitochondria between cells, rescues recipient cells from mitochondrial dysfunction and enhances cell viability^26^. Mitochondrial transfer is performed by isolating mitochondria from donor cells and transferring them to recipient cells of the nervous system^27^ or cellular senescence^28^. To isolate the mitochondria, cells are subjected to differential centrifugation to separate the mitochondrial and cytoplasmic fractions^29^. Isolated mitochondria are then transfected in vitro and in vivo using a variety of methods^30,31^, such as electroporation, fusion or co-culture^29,32^. Mitochondrial transfer can enhance mitophagy and inhibit mitochondrial oxidative stress^33–36^. Stem cells, such as mesenchymal stem cells, are good donors for mitochondrial transplantation^37,38^. Therefore, the utilization of stem cells as a source of mitochondria needs to be investigated.

Cardiomyocytes have a high density of mitochondria; they produce 90% of ATP, occupy 40% of the cardiac cell volume and are embedded in a dense and complex organization^39^, suggesting that they are promising donor cells for mitochondrial transplantation. Human pluripotent stem cells, such as embryonic stem (ES) cells and induced pluripotent stem (iPS) cells, can differentiate into functional cardiomyocytes^40^. Maturation of human ES-cell-derived cardiomyocytes increases the number and size of mitochondria and enhances the formation of mitochondrial lamellar cristae^41^, suggesting that iPS-cell-derived cardiomyocytes (iPSC-CMs) are a great source of healthy mitochondria. In the present study, we established iPS cell lines from healthy individuals and patients with BTHS and differentiated them to normal iPSC-CMs (normal CMs) and iPSC-CMs derived from patients with BTHS (BTHS CMs), which serve as mitochondrial donor cells and a disease model cardiomyocyte of BTHS, respectively. In this study, we aim to explore the effects of mitochondrial transfer from normal CMs to BTHS CMs on cardiac and mitochondrial functions in BTHS CMs and the molecular mechanism involved.

## Materials and methods

### Materials

mTeSR1 was purchased from Stem Cell Technology. Accutase (A1110501), Essential 8 (A1517001), trypsin–EDTA 0.25% (25200-056), trypsin–EDTA 0.05% (25300-054), TrypLE TM (12605-010), fetal bovine serum (16000-044), RPMI1640 no glucose (11879-020), DMEM/F12 (11330-032), mitoSOX (Red mitochondrial superoxide indicator; M36008, Thermo), tetramethylrhodamine (TMRM, T668) and Cytotune-iPS 2.0 Sendai Reprogramming Kit (A16517) were purchased from Thermo Fisher Scientific. Dulbecco's PBS (LB001-01) and RPMI1640 (LM011-01) were purchased from Welgene. Corning Matrigel hESC-Qualified Matrix LDEV-Free (354277) and Matrigel matrix GFR (354230) were purchased from Corning Life Sciences. Deoxyribonuclease 1 (D4263), fibronectin (F2006), albumin human (A9731), l-ascorbic acid 2-phosphate (A8960) and Y27632 (688000) were purchased from Sigma-Aldrich. CHIR99021 (S2924) and IWR-1-endo (72562) were purchased from Selleck Chemicals. l-Lactic acid (129-02666) was purchased from FUJIFILM Wako Chemical Industries. MitoFlamma Green (RMS1101) and MitoFlamma Deep Red, RMS1102) were purchased from BioActs.

### Analysis of genetic mutations in patients with BTHS

This study was approved by the Institutional Review Board of Pusan National University Yangsan Hospital (approval no. 05-2020-257). It was performed in accordance with the Declaration of Helsinki, and informed consent was obtained from all study participants. For targeted exome sequencing, pure genomic DNA was isolated from the participants’ leukocytes in the peripheral blood using the QIAamp DNA Blood Midi kit (Qiagen) according to the manufacturer’s standard protocols. The proband was applied to the TruSight One Sequencing Panel (Illumina), which included 125,395 probes targeting the 12-Mb region covering the exonic regions of 4813 genes with clinical implications. The probe was an 80-mer, which targeted libraries of nearly 500 bp, enriching 350–650 bases centered symmetrically at the midpoint of the probe. The captured target regions were sequenced using an Illumina HiSeq2500 sequencer (Illumina) according to the manufacturer’s instructions. Alignment and variant calling were performed using on-instrument software, followed by filtering and customized reporting of specific genes through analysis of the imported sequence data using the VariantStudio software. Variants in the dbSNP135 and TIARA databases for the Korean population and variants with minor allele frequencies of >0.5% in the 1000 Genomes database were excluded from further analyses. We selected only functional variants as pathogenic mutations. The functional annotation tools used were SIFT (http://sift.jcvi.org/), PROVEN (http://provean.jcvi.org/), PolyPhen2 (http://genetics.bwh.harvard.edu/pph2/) and MutationTaster (http://www.mutationtaster.org/). Mutation nomenclature was based on cDNA reference sequences for *TAZ* (NM_000116.3). Data on demographics and other clinical features were collected from the patients’ clinical records.

Sanger sequencing was used to confirm candidate variants and define the inheritance mode of the candidate variants via familial segregation testing. All candidate variants were sequenced bidirectionally using the ABI PRISM 3.1 Big Dye Terminator Kit (Applied Biosystems). The sequencing products were resolved on an ABI PRISM 3130XL sequencer (Applied Biosystems), and the chromatograms were analyzed using Sequencer 4.9 software (Gene Codes).

### Protein structural modeling

Crystal structures of the domains from wild-type *TAZ* were generated using SWISS-MODEL (https://swissmodel.expasy.org/). All structural images were generated using PyMOL, a molecular visualization software. The results from other predictive tools (Normal mode analysis (NMA)- and structure-based approaches) were also used to predict the effects of the mutation using the Dynamut web server with the normal mode analysis function (http://biosig.unimelb.edu.au/dynamut/).

### Isolation of urine-derived cells

This study protocol was approved by the Institutional Review Board at the Pusan National University Children’s Hospital in Yangsan, Gyeongsangnam-do, Republic of Korea (#L-2021-34; #PNU IRB/2021_25_BR). Urine-derived epithelial cells were isolated from urine derived from patients with BTHS. In brief, at least 130 ml urine was collected, centrifuged at 400*g* for 10 min and washed with PBS containing 100 U/ml penicillin, 100 µg/ml streptomycin and 500 ng/ml amphotericin B. Urine-derived cells were resuspended in the culture medium (DMEM/F-12 with 15 mM HEPES, 10% fetal bovine serum, 1% non-essential amino acids, 10 ng/ml recombinant human EGF, 36 ng/ml hydrocortisone, 5 µg/ml recombinant human insulin, 500 ng/ml epinephrine, 5 µg/ml human holo-transferrin, 4 pg/ml triodo-l-thyronine, 434.4 µg/ml alanyl-glutamine, 100 µg/ml penicillin–streptomycin, 2.5 µg/ml amphotericin B and 0.1% ROCK inhibitor) and seeded in gelatin-coated plates. When the cells were grown to 30% confluence, the cells were collected by incubation with trypsin–EDTA, seeded in a gelatin-coated plates and incubated at 37 °C in a humidified atmosphere with 20% O_2_ and 5% CO_2_.

### Reprogramming of urine-derived cells to iPS cells

To induce reprogramming of urine-derived cells to iPS cells, urine-derived cells at passages below 5 were seeded in a 24-well gelatin-coated plate for 1 day and then treated with the CytoTune-iPS 2.0 Sendai reprogramming kit according to the manufacturer’s instructions. After Sendai virus infection, the cells were transferred to Matrigel (10 μg/cm^2^)-coated 12-well plate and cultured with Essential 8 medium (Thermo Fisher Scientific). After iPS cell colonies emerged, the first four passages were carried out mechanically to specifically isolate colonies with an iPS cell morphology. The iPS cell colonies were maintained on Matrigel-coated plates in Essential 8 medium for more than 20 passages before being used in experiments and cell banking. The number and arrangement of chromosomes in iPS cells were analyzed by standard G-banded karyotype analysis at Dx&Vx.

### Cardiomyocyte differentiation of iPS cells

Cardiomyocyte differentiation was performed as previously reported with slight modification^41^. In brief, iPS cells were seeded on Matrigel-coated plates in Essential 8 medium and differentiated to cardiomyocytes by treatment with 10 μM CHIR99021, 50 ng/ml Activin A and 50 μg/ml l-ascorbic acid for 24 h in RPMI medium supplemented with B27 minus insulin. The next day, cells were treated with 5 μM IWR1-endo and 50 μg/ml l-ascorbic acid for 48 h, followed by treatment with 5 μM IWR1-endo until day 6. On day 7, the cells were cultured with RPMI supplemented with B27 for 48 h. iPSC-CMs were further enriched by metabolic selection with glucose-free culture medium supplemented with 4 mM sodium l-lactate (Sigma-Aldrich) at day 10. After the metabolic selection, iPSC-CMs were reseeded onto Matrigel-coated plates in Essential 8 medium supplemented with 10 μM Y27632 and maintained at 37 °C and 5% CO_2_ by replacing culture media every 2 days.

### Mitochondrial transplantation

The mitochondria were isolated from donor-derived iPSC-CMs on day 30. Approximately 5 million cells were collected in a 1.5-ml tube, resuspended in mitochondrial isolation buffer (25 mM Tris–HCl (pH 7.4), 250 mM sucrose and 1 mM EDTA) and disrupted by passing through a 23 G syringe 20 times. The cells were centrifuged at 1,000*g* for 10 min to separate nuclei and cell debris, and the supernatant was transferred to a new tube and centrifuged at 9,000*g* for 10 min. The precipitated mitochondrial fractions were resuspended in the mitochondrial isolation buffer, and the protein concentration of mitochondrial fractions was determined using the Bradford assay. For mitochondrial transplantation, recipient BTHS CMs were treated with the purified mitochondrial fractions (5 pg mitochondrial proteins per recipient cell) in the cardiomyocyte culture medium for indicated time periods and replaced with fresh culture medium. To measure the efficacy of mitochondrial transplantation, mitochondria in normal CMs and BTHS CMs were stained with MitoFlamma Green and MitoFlamma Deep Red, respectively, for 30 min at 37 °C and 5% CO_2_ in an incubator, followed by mitochondrial isolation and transplantation in vitro. After mitochondrial transplantation, the intensity and intracellular distribution of MitoFlamma Green and MitoFlamma Deep Red fluorescences in BTHS CMs were analyzed using fluorescence-activated cell sorting (FACS) or confocal microscope using a Zeiss LSM 980 microscope. In addition, we analyzed the levels of mitochondrial perimeter and area stained with MitoFlamma in ImageJ.

### Immunocytochemistry

Normal CMs and BTHS iPSC-CMs were seeded in confocal dishes, fixed in 4% paraformaldehyde and permeabilized with 0.1% Triton X-100 in PBS buffer for 20 min. The cells were incubated with the primary antibodies (Supplementary Table 1) at 4 °C for 1 h and washed three times with 0.1% Triton X-100 in PBS buffer, followed by incubation with the secondary antibodies for 1 h. After washing three times, the samples were mounted with ProLong Antifade Mountants containing 4′,6-diamidino-2-phenylindole (DAPI) for immunocytochemistry analysis using a confocal microscope.

### Quantitative real-time PCR

To extract total RNA from iPSC-CMs, the cells were lysed using TRIzol reagent according to the manufacturer’s instructions. The extracted RNA was reverse-transcribed using oligo-dT primers and Superscript II. For real-time quantitative real-time PCR, we used the SYBR Green PCR Master Mix, and the reactions were performed on a Quant Studio 3 Real-time PCR System (Applied Biosystems). Gene-specific primers (Supplementary Table 2) were used for PCR amplification. Target gene expression was normalized to endogenous GAPDH using the comparative cycle time method.

### Western blot analysis

Cells were lysed in a lysis buffer (Tris–HCl, EDTA, EGTA, NaCl, Na_3_VO_4_, phenylmethylsulfonyl fluoride, sodium pyrophosphate, β-glycerol phosphate and Triton X-100 at pH 7.4). After quantification of protein concentrations of the cell lysates using Bradford assay, the cell lysates were solubilized in Laemmli SDS sample buffer for western blotting. Proteins in cell lysates were separated by SDS–PAGE and transferred to a nitrocellulose or polyvinylidene fluoride membrane. Membranes were blocked with 5% nonfat milk for 1 h and incubated overnight with the primary antibodies (1:1,000 dilution), followed by incubation with secondary antibodies (1:5,000 dilution) for 2 h. The membrane was developed using the ECL western blotting system (GE-RPN2106), and the images were captured using the ImageQuant 800 western blot imaging system (GE Healthcare) and quantified using ImageJ, an image processing and analysis software developed by the National Institutes of Health.

### Measurement of mitochondrial membrane potential and ROS

iPSC-CMs were seeded in confocal dishes and allowed to stabilize in the growth medium for 24 h. To measure mitochondrial membrane potential or reactive oxygen species (ROS), the cells were treated with 50 µM TMRM or 2 µM mitoSOX, respectively, for 30 min at 37 °C. For labeling of mitochondria, the cells were counterstained with 1 mM mitoFlamma (Green and Deep Red). After incubation with the fluorescent probes, the cells were washed twice with PBS, and the fluorescence signals were analyzed using a Zeiss LSM 900 microscope. The levels of TMRM and superoxide in mitochondria were measured by ImageJ software.

### TEM analysis

The samples were fixed in 2% paraformaldehyde and 2.5% glutaraldehyde in 0.15 M cacodylate buffer (pH 7.4) overnight in a 4 °C refrigerator. After rinsing with cacodylate buffer, the sections were post-fixed in 1% osmium tetroxide for 2 h, dehydrated using a graded series of ethanol solutions and embedded in epoxy resin. Next, 100-nm-thin sections were cut using an ultramicrotome, stained with uranyl acetate and lead citrate, and examined using a transmission electron microscope at an accelerating voltage of 120 kV. High-speed transmission electron microscopy (TEM; TECNAI G2) was supported by the Brain Research Core Facilities of KBRI.

### Measurement of mitochondrial respiration

Mitochondrial respiration was measured using the Mito Stress Test Kit on an XFe 96 analyzer. Approximately 50,000 cells were seeded in GFR Matrigel-coated 96-well plates and allowed to attach to 90% confluence in the growth medium. The Seahorse XF Cell Mito Stress Test Kit User Guide (Agilent Technologies) was used for this assay. The oxygen consumption rate was measured while treating the cells with 1.5 μM oligomycin, 0.5 μM FCCP, 1 μM rotenone and 1 μM Antimycin A. A Seahorse XF Mito Stress Test report generator was used to automatically calculate these parameters from the wave data.

### Measurement of electrophysiological function

The electrical function of the iPSC-CMs was measured using a Maestro Edge multiwell microelectrode array. Electrode plates coated with fibronectin (50 μg/ml) were seeded with iPSC-CMs at a density of 5 × 10^4^ cells and incubated at 37 °C and 5% CO_2_ in an incubator for 3 days. The Maestro Edge was used to record key parameters such as action potential, field potential, propagation and contractility. The number of cells was recorded every 2 days after stabilization.

### Statistical analysis

All statistical analyses were performed using GraphPad Prism. One-way analysis of variance (ANOVA), Student’s *t*-test and Pearson correlation analysis used can be found in the figure captions. Nonsignificant comparisons were excluded from the graphs. Unless stated otherwise, multiple comparisons were performed between the experimental groups and control conditions. All experiments were performed independently at least twice, and the results are representative.

## Results

### Clinicopathological characteristics and genetic alterations in a patient with BTHS

A 2-day-old boy was referred to our metabolic clinic for the genetic evaluation of an unknown cause of cardiomyopathy. He was born at 39 weeks and 3 days of gestation and was 2.93 kg at birth. Prenatal ultrasonography revealed an enlarged heart. On the day of birth, the infant received ventilator care for tachypnea and desaturation after delivery. Chest radiography showed cardiomegaly. Echocardiography revealed that the ejection fraction in the left ventricle was greatly reduced to 18–20% and the cardiomyopathy was dilated at that time (Supplementary Fig. 1). The patient’s B-type natriuretic peptide and creatine kinase levels increased to 5,000 pg/ml and 1,339 (reference 0–171) U/l, respectively. Urinary organic acid analysis revealed an elevated 3-methylglutaric acid level of 2.2 (reference <0) mmol/mol Cr and 3-methylglutaconic acid level of 18.5 (reference <9.0) mmol/mol Cr. The patient is currently waiting for a heart transplant because he is taking heart failure drugs, including enaprin and dilatrend, but there is no treatment for the underlying issue.

**Fig. 1:**
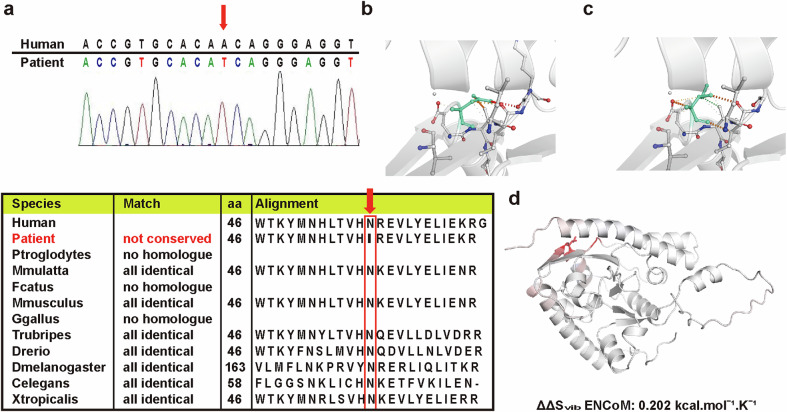
The patient’s genetic test result and pathogenicity of the variant. **a** Sanger sequencing confirmed a novel heterozygous novel variant, c.137A>T (p.Asn46Ile) (NM_000116.3) in TAFAZZIN, which had been identified by targeted exome sequencing of the patient’s genome, which is indicated by the red arrow. Alignment of the predicted amino acid sequence of TAFAZZIN among different species is indicated by the dotted red box. Sequences were aligned with blastp (https://blast.ncbi.nlm.nih.gov/). **b** Wild-type and **c** mutant residues (p.Asn46Ile) in *TAZ* are colored light green and represented as sticks alongside the surrounding residues, which are involved in any type of interaction. Blue dots represent halogen bonds, red dots represent hydrogen bonds and orange dots represent weak hydrogen bonds. The crystal structure of the domain from wild-type TAZ was generated by SWISS-MODEL (https://swissmodel.expasy.org/) and is depicted as a cartoon representation. **d** Results from other predictive tools (NMA-based and other structure-based approaches) displayed to predict the mutation effect using the Dynamut web server with the normal mode analysis function (http://biosig.unimelb.edu.au/dynamut/). A visual representation of the Δ vibrational entropy energy is shown in which the amino acids are colored according to the vibrational entropy change upon mutation. Red regions indicate a gain in flexibility.

We performed a targeted exome sequencing on the patient and identified a novel missense variant, c.137A>T (p.Asn46Ile), which was an inherited genetic mutation from the patient’s mother. This novel variant was identified as a rare variant that had not been previously reported in the general population (gnomAD, KRGDB). The p.Asn46Ile mutant of *TAZ* is characterized by a base substitution of A to T at nucleotide 137. This sequence is completely conserved across multiple species (Fig. 1a). Protein crystallization revealed that the p.Asn46Ile variant may affect the formation of hydrogen bonds between the surrounding residues (Fig. 1b, c). Besides, the p.Asn46Ile variant was affecting the vibration-related entropy change upon mutation, increasing molecule flexibility (ΔΔSvib ENCoM: 0.202 kcal/mol/K), leading to protein destabilization, based on structure-based predictions (Fig. 1d).

### Establishment of patient-derived iPS cells and differentiation to cardiomyocytes

To establish normal and BTHS iPS cells, we isolated urine epithelial cells from a healthy donor and a patient with BTHS and generated colony-forming iPS cells through Sendai virus-mediated overexpression of OSKM genes (Supplementary Fig. 2a). These iPS cells exhibited pluripotency with the ES-cell-like morphology of a round, irregular, flat shape and were densely packed with a smooth and glossy surface. Immunocytochemistry and FACS analysis confirmed the expression of pluripotency markers, including OCT4, NANOG, SOX2, LIN28A and SSEA4, in the iPS cells (Supplementary Fig. 2b,c). The BTHS iPS cells exhibited normal chromosome number and karyotype structure but harbored the p.Asn46Ile mutation in the *TAZ* gene (Supplementary Fig. 3).

**Fig. 2:**
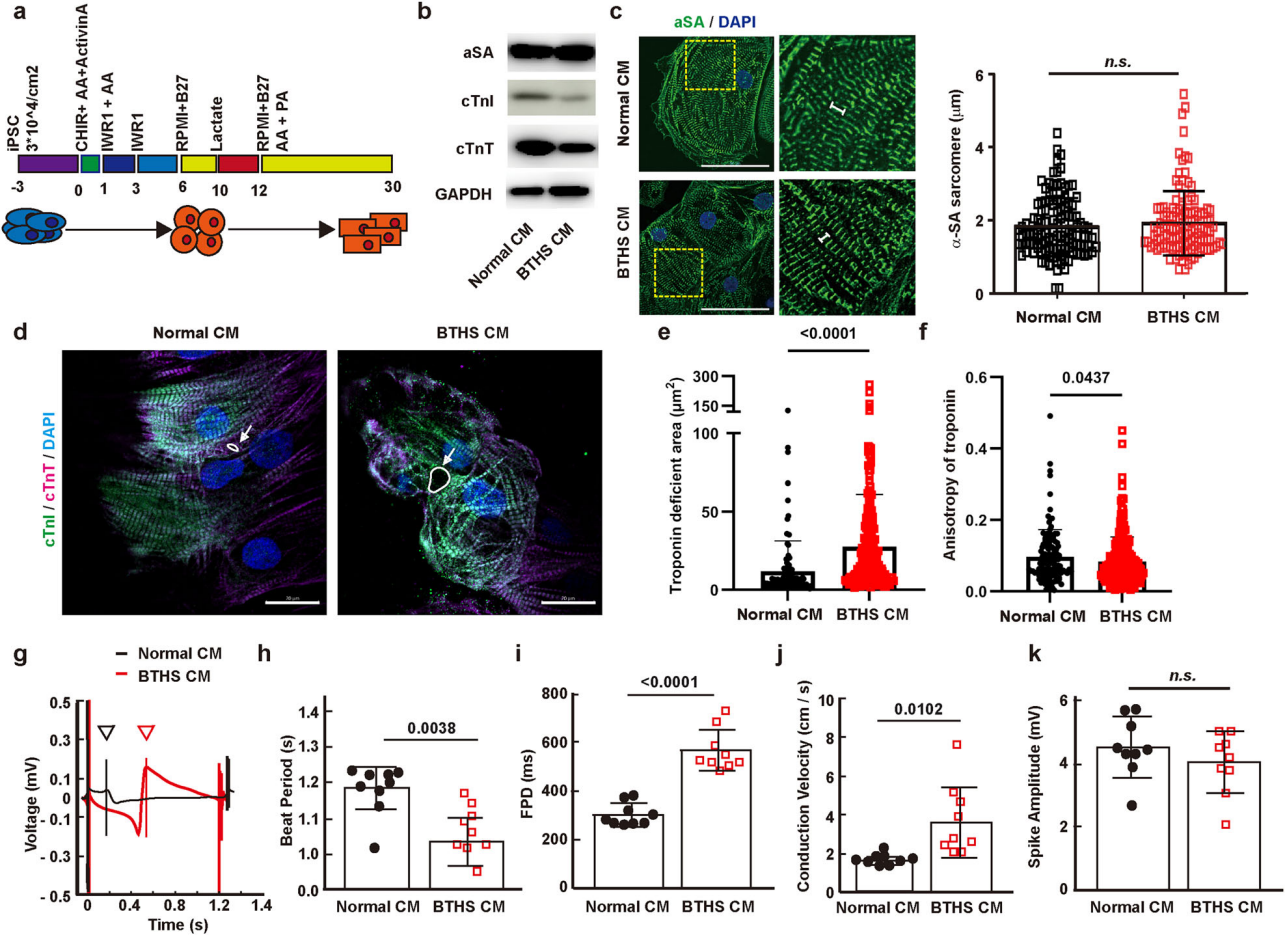
Characterization of cardiomyocytes derived from normal and BTHS iPS cells. **a** A schematic diagram of cardiomyocyte differentiation from iPS cells. **b** The expression of cardiomyocyte and mitochondrial markers in normal and BTHS CMs at day 30. **c** Measurement of α-SA sarcomere length in immunocytochemistry on 63× magnification observation. Data are presented as mean ± s.d. (*n* = 6, biological statistics; *n* = ~83–185, technical statistics). Student’s *t*-test. n.s., not significant. **d** Immunocytochemistry of cTnT, cTnI and DAPI on day 30. Scale bar, 20 μm. Normal CMs and BTHS CMs were stained with anti-cTnT antibody (red color), anti-cTnI antibody (green color) and DAPI (nucleus staining; blue color), followed by analysis with confocal microscopy. The troponin-deficient area is indicated by the white arrows. **e** The cardiomyocyte marker troponin-deficient area was measured using ImageJ. *n* = ~108–223. **f** Anisotropy of cardiac troponin measured using ImageJ in **d**. *n* = 118–220. **g** A representative plot of the FPD in normal (black) and BTHS CMs (red) at day 30. **h** Mean of beat period. **i** Mean of FPD. **j** Conduction velocity. **k** Mean of spike amplitude. These are parameters of field potential values of **g**. The values are statistically measured through unpaired *t*-test with Welch’s correction. Biological repeat *n* = 9. Data are presented as mean ± s.d. n.s., not significant. Statistical significance was calculated using an unpaired, two-tailed *t*-test, and *P* values are indicated. iPSC, iPS cell.

To establish a BTHS disease model, normal iPS cells and BTHS iPS cells were differentiated into normal CMs and BTHS CMs using a cardiomyocyte differentiation protocol (Fig. 2a). The expression of cardiac structural markers, including cardiac troponin I (cTnI), cardiac troponin T (cTnT) and α-sarcomeric actinin (α-SA), was analyzed on day 30 after inducing cell differentiation. The expression levels of cTnI and cTnT were reduced in BTHS CMs compared with those in normal CMs (Fig. 2b), while the expression level of α-SA and the length of α-SA-positive sarcomere were not significantly different between normal CMs and BTHS CMs (Fig. 2b,c). Immunostaining for cTnT and cTnI showed that the sarcomere structures irregularly connected in BTHS CMs (Fig. 2d). Furthermore, BTHS CMs exhibited increased troponin-deficient punctate area in cytosol and reduced anisotropic alignment of troponin (Fig. 2e, f), suggesting that BTHS CMs have an immature cardiac structure compared with normal CMs.

To compare the electrical function of normal CMs and BTHS CMs, multielectrode arrays were used to measure the field potential durations (FPDs; Fig. 2g). BTHS CMs exhibited a shorter beat period than normal CMs, indicating a faster beating rate (Fig. 2h). Furthermore, BTHS CMs exhibited a longer FPD and conduction velocity than normal CMs, while spike amplitudes did not change significantly in BTHS CMs (Figs. 2i–k). These results suggest that BTHS CMs exhibit abnormal phenotypic and cardiac electrophysiological properties that may contribute to the clinical symptoms of BTHS, such as heart failure, long-QT syndrome, arrhythmia and sudden cardiac death.

### Abnormal mitochondrial function and biogenesis in BTHS CMs

**Fig. 3:**
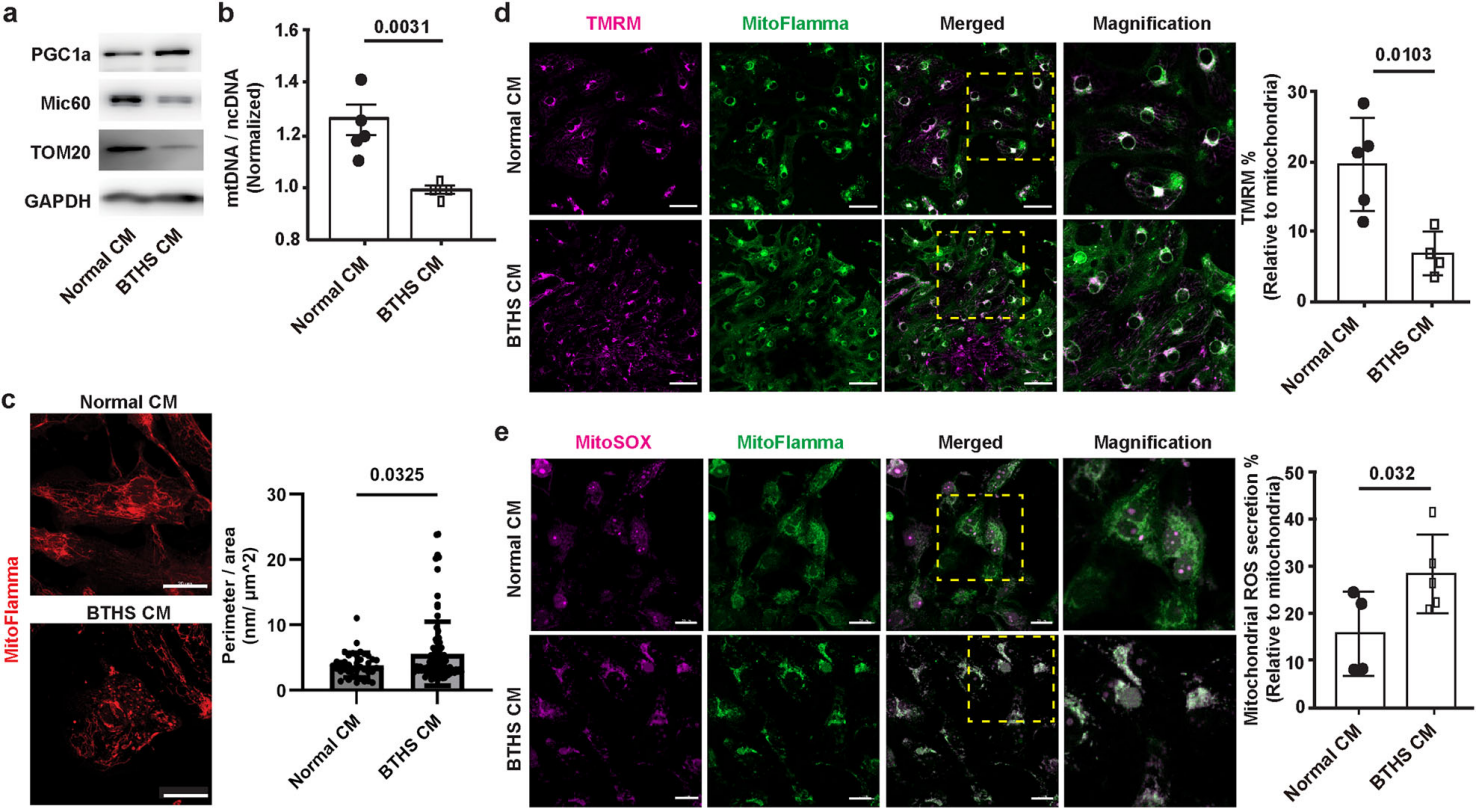
Mitochondrial dysfunction and reduced mitochondrial biogenesis in BTHS CMs. **a** The expression of mitochondrial proteins in normal and BTHS CMs. Western blotting was used to determine the protein levels of mitochondrial markers (PGC-1α, MIC60 and TOM20) and GAPDH. **b** Reduced mitochondrial DNA levels in BTHS CMs. ncDNA and mtDNA were quantified, and the ratio of mtDNA/ncDNA is shown (*n* = 5). **c** Decreased length and increased perimeter of mitochondria in BTHS CMs. Left: mitochondria were stained with MitoFlamma Deep Red in normal and BTHS CMs, and mitochondrial morphology was analyzed by confocal microscopy. Scale bar, 20 μm. Right: the mitochondrial perimeter was determined using ImageJ. Data are presented as mean ± s.d. (*n* = ~39–91). **d** Mitochondria and mitochondrial membrane potential were probed with MitoFlamma Green and TMRM dyes in normal and BTHS CMs at day 30 after inducing cell differentiation, and merged images are shown. Scale bar, 50 μm (*n* = 5). **e** Enhanced mitochondrial ROS in BTHS CMs. Normal and BTHS CMs were double stained with MitoSOX and MitoFlamma Green, and merged images are shown. Scale bar, 20 μm. Data are presented as mean ± s.d. (*n* = 4 ~ 5). Statistical significance was calculated using an unpaired, two-tailed *t*-test, and *P* values are indicated.

To explore whether mitochondrial structure and function were altered in BTHS CMs, the expression levels of mitochondrial proteins were determined using western blotting. BTHS CMs exhibited reduced expression levels of MIC60, an integral protein of the mitochondrial inner membrane, and TOM20, a mitochondrial outer membrane protein, compared with normal CMs, while the expression of peroxisome proliferation-activated receptor-γ coactivator-1α (PGC-1α), a mitochondrial biogenesis-activating protein, was increased (Fig. 3a). Despite the increased expression of PGC-1α, the mitochondrial copy number, which was indicated by mitochondrial DNA (mtDNA)/nuclear DNA (ncDNA), was decreased in BTHS CMs (Fig. 3b), suggesting reduced amounts of mitochondria in the BTHS CMs. Furthermore, we found that mitochondria in BTHS CMs exhibited a fragmented phenotype, and the perimeter of mitochondria was increased in BTHS CMs compared with that in normal CMs (Fig. 3c).

To explore whether mitochondrial capacities were altered in BTHS CMs, we measured the mitochondrial membrane potential and superoxide production using TMRM and MitoSOX as fluorescent probes. The TMRM-positive mitochondrial membrane potential of BTHS CMs was lower than that of the normal CMs (Fig. 3d). Furthermore, the MitoSOX-positive fluorescence in BTHS CMs was higher than that in normal CMs (Fig. 3e). Reduced mitochondrial membrane potential and increased mitochondrial superoxide production indicate mitochondrial dysfunction in BTHS CMs.

### Allogenic mitochondrial transplantation improves mitochondrial function in BTHS CMs

**Fig. 4:**
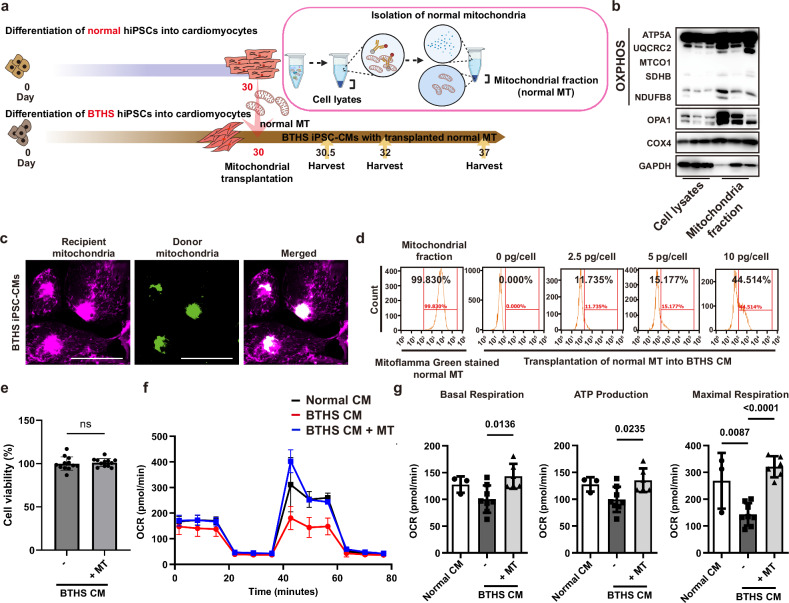
Allogenic transplantation of mitochondria isolated from normal CMs to BTHS CMs. **a** A schematic diagram of transplantation schedule of mitochondria into BTHS CMs and isolation method of mitochondria from normal CMs. **b** Western blot analysis of cytosolic and mitochondrial fractions isolated from normal CMs. The expression of mitochondrial proteins (OPA1, COX4 and OXPHOS system (ATP5A, UQCRC2, MTCO1, SDHB and NDUFB8)) and cytosolic marker (GAPDH) were determined by western blotting. Triplicated experiments are shown. **c** Tracking of transplanted mitochondria in the recipient BTHS CMs. Mitochondria of BTHS CMs (red color) and normal CMs (green color) were labeled with MitoFlamma Deep Red and MitoFlamma Green, respectively. The recipient BTHS CMs were treated with mitochondria (green color) isolated from normal CMs. Co-localization of donor and recipient mitochondria is shown in a merged image. Scale bar, 50 μm. **d** Dose-dependent effects of mitochondrial transplantation. Mitochondria in normal CMs were labeled with MitoFlamma Green, isolated and dose-dependently treated to BTHS CMs. The levels of normal mitochondria transplanted into BTHS CMs were determined by FACS analysis, and the percentages of MitoFlamma Green-positive BTHS CMs are indicated. **e** BTHS CMs transplanted with mitochondria at a concentration of 5 pg per cell were assessed for cell viability using the WST-8 assay 1 week after transplantation. **f** Effects of mitochondrial transplantation on OXPHOS in BTHS CMs. The oxygen consumption rate (OCR) and extracellular acidification rate (ECAR) were recorded for 90 min. **g** Effects of mitochondrial transplantation on basal respiration, ATP production and maximal respiration. Statistical significance was calculated using one-way ANOVA with Holm–Šidák’s multiple-comparisons test, and *P* values are shown. The graphic diagram was created with BioRender.com.

To explore whether allogeneic mitochondrial transplantation from normal CMs to BTHS CMs could restore mitochondrial function, we isolated mitochondria from normal CMs and treated them to BTHS CMs (Fig. 4a). Western blot analysis showed that the mitochondrial fractions contained higher levels of mitochondrial proteins including MIC60, TOM20 and OPA1, and oxidative phosphorylation (OXPHOS) system (ATP5A, UQCRC2, MTCO1, SDHB and NDUFB8) than cell lysis fractions (Fig. 4b and Supplementary Fig. 4a). The mitochondria isolated from normal CMs and BTHS CMs contained similar protein concentrations (Supplementary Fig. 4b). To track transplanted mitochondria, normal CMs were fluorescently labeled with MitoFlamma Green. One week after fluorescence labeling, 55.7% of MitoFlamma Green fluorescence in normal CMs persisted (Supplementary Fig. 5a), suggesting long-lasting mitochondrial fluorescence labeling by 1 week. MitoFlamma Green-labeled mitochondria were isolated from normal CMs and treated to the recipient BTHS CMs. Before mitochondrial transplantation, the endogenous mitochondria of recipient BTHS CMs were prestained with MitoFlamma Deep Red dye. After mitochondrial transplantation, MitoFlamma Green-labeled exogenous mitochondria colocalized with MitoFlamma Deep Red-positive endogenous mitochondria in BTHS CMs (Fig. 4c). Increasing the doses of mitochondria during the transplantation process augmented the percentages of MitoFlamma Green-positive BTHS CMs in FACS analysis (Fig. 4d), without affecting cell viability of BTHS CMs (Fig. 4e). One week after the transplantation of normal mitochondria into BTHS CMs, the fluorescence of the transplanted mitochondria decreased to approximately 13% and disappeared 2 weeks after transplantation (Supplementary Fig. 5b), suggesting that transplanted mitochondria could be detected up to 1 week after transplantation.

Cardiolipin deficiency in the heart of patients with BTHS results in abnormal mitochondrial structure, which causes an increase in mitochondrial superoxide and a decrease in mitochondrial respiration^42^. To explore the effects of mitochondrial transplantation on BTHS-associated mitochondrial dysfunction, mitochondrial superoxide levels were quantified using MitoSOX, a fluorescent probe specific for mitochondrial superoxide. BTHS CMs exhibited higher MitoSOX-positive fluorescence than normal CMs, and transplantation of normal mitochondria reduced mitochondrial superoxide levels to levels similar to those in normal CMs (Supplementary Fig. 6). To explore the effects of allogeneic mitochondrial transplantation on mitochondrial function, the cellular oxygen consumption rates were measured. BTHS CMs significantly reduced maximal respiration. Twelve hours after mitochondrial transplantation, the basal respiration rate, maximum respiration and ATP production of BTHS CMs increased similarly to those of normal CMs (Figs. 4f, g), suggesting that allogeneic mitochondrial transplantation enhanced the mitochondrial respiration ability in BTHS CMs.

### Mitochondrial transplantation induces mitophagy in BTHS CMs

**Fig. 5:**
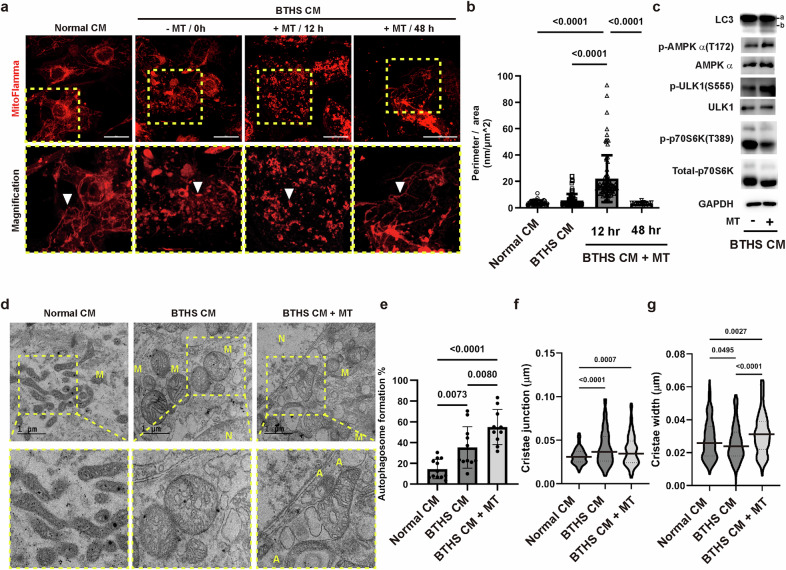
Effects of mitochondrial transplantation on mitophagy in BTHS CMs. **a** Effects of mitochondrial transplantation on mitochondrial morphology in BTHS CMs. Top: normal and BTHS CMs were stained with MitoFlamma (Deep Red) at the indicated times after mitochondria transplantation. Bottom: magnified images of the dotted area are shown. Arrowheads indicate mitochondria. Scale bar, 20 μm. **b** The mitochondrial perimeter was measured using ImageJ software. Data are presented as mean ± s.d. (*n* = ~39–91). **c** Effects of mitochondrial transplantation on mitophagy and autophagy in BTHS CMs. After transplantation of normal mitochondria into BTHS CMs, the expression and the phosphorylation levels of LC3, AMPKa, ULK, p70S6K and GAPDH were determined by western blot analysis 48 h after transplantation. **d** Effects of mitochondrial transplantation on mitochondrial morphology in BTHS CMs. Mitochondrial morphology of normal CMs, BTHS CMs and mitochondria-transplanted BTHS CMs analyzed by TEM analysis. Mitochondria (M) and autophagosome (A) are indicated. Scale bar, 1 μm. **e** Quantification of mitochondria fused with autophagosomes in TEM images. Data are presented as mean ± s.d. (*n* = ~10–12). **f**, **g** Cristae junction (**f**) and width (**g**) in the TEM images were measured by ImageJ software. Data are presented as mean ± s.d. (*n* = ~125–231). Statistical significance was calculated using one-way ANOVA with Holm–Šidák’s multiple-comparisons test, and *P* values are shown in the figures.

Exogenously transplanted mitochondria can mix with recipient mitochondria via mitochondrial fusion and fission. The mitochondrial perimeter of BTHS CMs was measured to determine the effects of mitochondrial transplantation on mitochondrial structure (Fig. 5a). Twelve hours after transplantation, the mitochondria had a longer mitochondrial perimeter and a more fragmented structure than the parental BTHS CMs, whereas at 48 h, the mitochondrial perimeter was reduced to the values of the normal CMs. These results indicated that mitochondrial turnover occurred within 48 h after mitochondrial transplantation (Fig. 5a, b). Mitophagy is an autophagy-mediated process that selectively removes damaged mitochondria^15^. To explore whether mitophagy is involved in the mitochondrial transplantation-induced restoration of mitochondrial functions, we analyzed the expression levels of proteins involved in autophagy and mitophagy. Mitochondrial transplantation increased the protein levels of LC3b, p-AMPKα and p-ULK1 in BTHS CMs (Fig. 5c and Supplementary Fig. 7), whereas mitochondrial transplantation attenuated the level of p-p70S6 kinase, which has been known to inhibit autophagy^43^, in BTHS CM (Fig. 5c). The levels of mitochondrial markers (MIC60, TOM20 and VDAC) and autophagy markers (ATG5, glycosylated lysosomal associated membrane protein 1 (LAMP1) and LC3b) were significantly increased 1 week after mitochondrial transplantation (Supplementary Fig. 8), indicating induction of mitophagy activation in the week after mitochondrial transplantation. These results suggested that exogenously transplanted normal mitochondria stimulated mitophagy in BTHS CMs.

To further clarify the effects of mitochondrial transplantation on mitophagy in iPSC-CMs, we performed TEM image analysis. In BTHS CMs, the mitochondria exhibited an abnormal morphology (Fig. 5d), including shortened mitochondria binding to lysosomes for degradation, autophagosome-like structures and appearances resembling transplanted normal mitochondria (Supplementary Fig. 9). Mitochondrial transplantation increased the number of autophagic vacuoles containing damaged mitochondria and induced the fusion of endogenous and transplanted mitochondria. The number of damaged mitochondria coexisting within autophagic vacuoles with matrix edema and collapsed cristae was higher in BTHS CMs transplanted with normal mitochondria (Fig. 5e). Furthermore, when the cristae junctions were measured at the beginning and end of the cristae, BTHS CMs transplanted with normal mitochondria showed no change in the cristae junctions but a wider width in the center of the cristae (Fig. 5f, g). These results suggest that mitochondrial transplantation induces mitophagy in BTHS CMs.

### Mitochondrial transplantation stimulates maturation of BTHS CMs

**Fig. 6:**
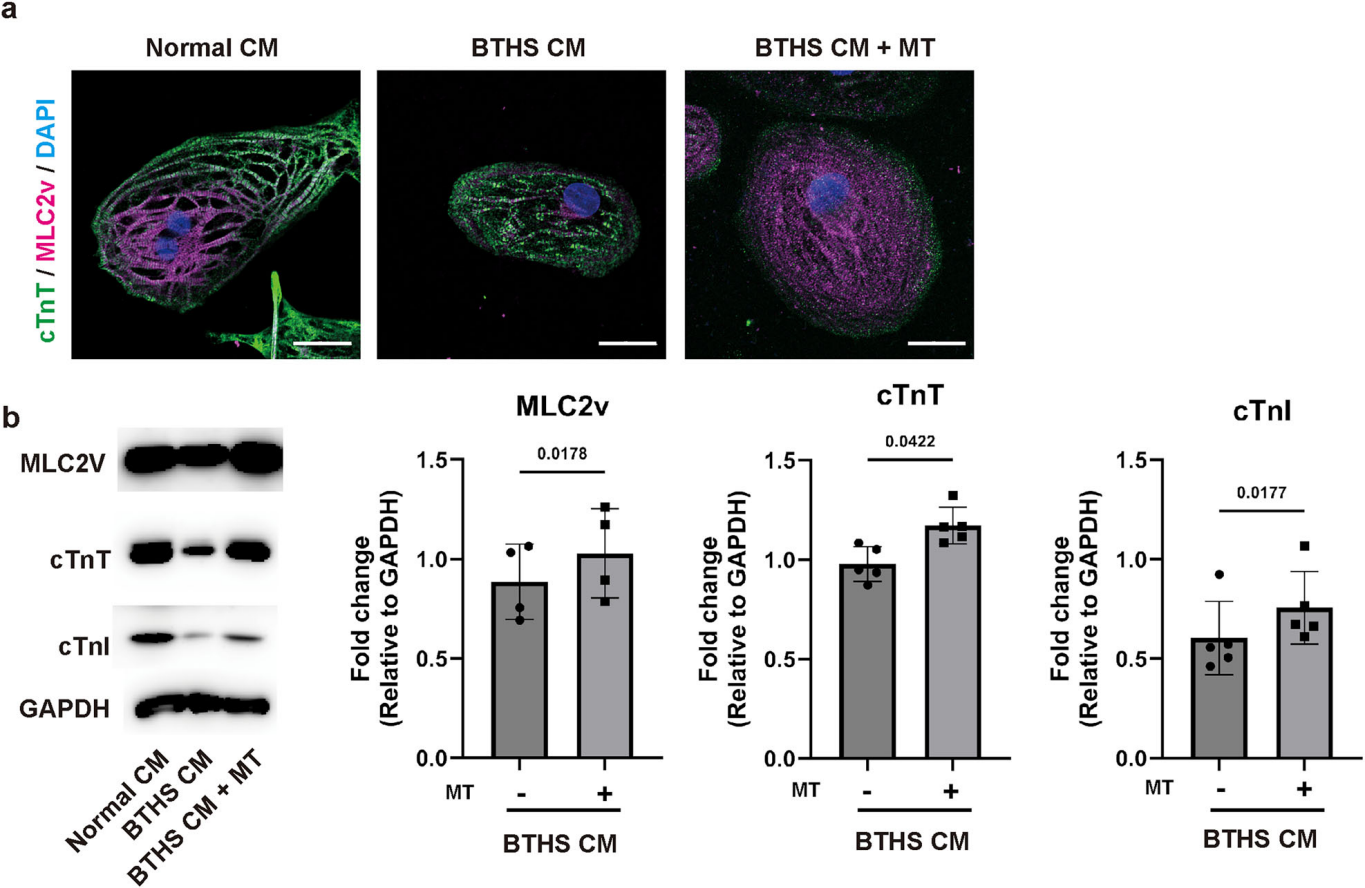
Effects of mitochondrial transplantation on expression of cardiac structure markers. **a** Immunostaining of cTnT and MLC2v in normal CMs, BTHS CMs and BTHS CM + MT. Normal CMs, BTHS CMs and BTHS CMs transplanted with MT (48 h after transplantation) were stained with anti-cTnT antibody (green color), anti-MLC2v antibody (red color) and DAPI (blue color), respectively. Scale bar, 20 μm. **b** Effects of mitochondrial transplantation on expression of cardiac markers. Left: the expression levels of cardiac markers (α-SA, cTnT and cTnI) and GAPDH were determined by western blotting in normal CMs, BTHS CMs and BTHS CM + MT. Right: the protein band intensities were quantified using ImageJ software, and data are presented as mean ± s.d. (*n* = ~4–5). Statistical significance was calculated using one-way ANOVA with Holm–Šidák’s multiple-comparisons test, and *P* values are shown in the figures.

To explore the effects of mitochondrial transplantation on the cardiomyocyte structure, we examined the expression of cardiomyocyte-specific structural proteins. Compared with normal CMs, BTHS CMs exhibited lesser expression of MLC2v, a mature CM marker, and irregular and disorganized connectivity of cTnT-positive sarcomere. Transplantation of normal mitochondria led to increased expression of cTnT, cTnI and MLC2v in BTHS CMs (Fig. 6a, b), suggesting that allogeneic transplantation of mitochondria isolated from normal CMs stimulates the cardiac maturation of BTHS CMs.

**Fig. 7:**
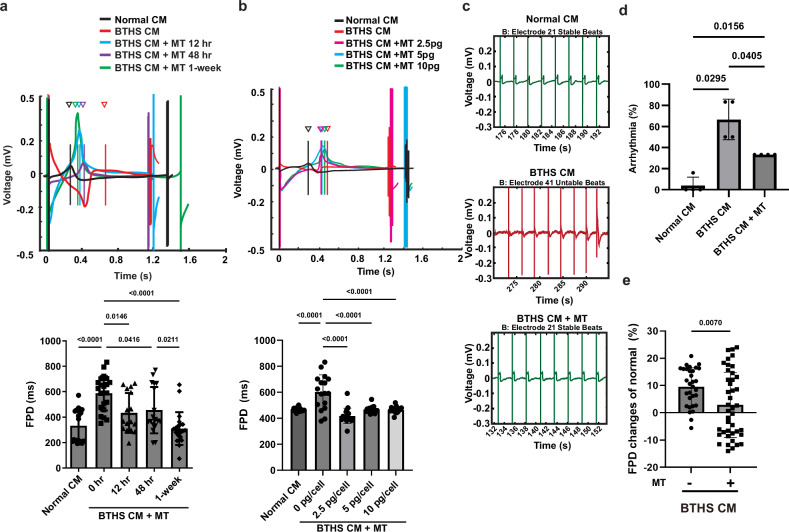
Effects of mitochondrial transplantation on electrophysiology of BTHS CMs. **a** The field potentials of cardiomyocytes were analyzed using the Axion cardiac analysis tool. Representative plot of field potential and the duration records 12 h, 48 h and 1 week after normal mitochondrial transplantation in BTHS CMs. **b** A representative plot of field potential and the duration records after normal mitochondrial transplantation with concentrations of 2.5, 5 and 10 pg per cell in BTHS CMs for 1 week after transplantation. **c** Representative beat flow, including arrhythmias, at the electrodes during normal, BTHS and iPSC-CM transplantation with normal mitochondria after 1 week. **d** Calculation of the frequency of arrhythmias by measuring microelectrode array (MEA) in normal CMs, BTHS CMs and BTHS CM + MT (1 week after transplantation). **e** Effects of mitochondrial transplantation on the FPD of BTHS CMs. After transplantation of normal CM-derived mitochondria into BTHS CMs, the relative FPD values of BTHS CMs compared with those of normal CMs are shown. This analysis was produced by the Axion cardiac analysis tool and metric plotting tool. Data are presented as mean ± s.d. (*n* = ~10–11). Statistical significance was calculated using one-way ANOVA with Holm–Šidák’s multiple-comparisons test, and *P* values are shown in the figures.

Next, we investigated the effect of mitochondrial transplantation on the electrophysiological function of BTHS CMs. BTHS CMs exhibited higher FPD than normal CMs, and mitochondrial transplantation time-dependently decreased the FPD values (Fig. 7a). One week after the mitochondrial transplantation, the FPD values of BTHS CMs recovered to values similar to those of normal CMs. Furthermore, mitochondrial transplantation into BTHS CMs reduced the FPD values with the maximal effect at the experimental condition treated with 2.5 pg mitochondria per BTHS CM (Fig. 7b). Compared with normal CMs, BTHS CMs exhibited increased cardiac arrhythmias, and mitochondrial transplantation significantly reduced the arrhythmias number 1 week after mitochondrial transplantation (Fig. 7c, d). In BTHS CMs, the FPD value, which represents the QT interval, was prolonged, suggesting long-QT syndrome. However, 1 week after transplantation of normal mitochondria, the prolonged FPD value of BTHS CMs decreased similarly to that of normal CMs (Fig. 7e). These results suggest that mitochondrial transplantation alleviates cardiac dysfunction in BTHS CMs.

## Discussion

The patient with BTHS exhibited dilated cardiomyopathy before birth and required ventilator treatment and critical care due to cardiac dysfunction immediately after birth. Severely dilated cardiomyopathy has also been reported in patients with BTHS; however, the molecular mechanisms underlying fetal cardiomyopathy or cardiac arrhythmia in early BTHS embryos remain elusive^3,11,17^. Similar to the patient’s clinical results, BTHS CMs exhibited abnormal cardiac electrical properties, which may contribute to BTHS-associated symptoms such as heart failure, long-QT syndrome, arrhythmia and sudden cardiac death. It has been reported that *TAZ* mutations contribute to trabeculated myocardium and left ventricular noncompaction^44^. BTHS CMs modeled with *TAZ* gene modifications exhibit lower-than-normal sarcomere organization and poor mitochondrial respiratory function^21^. Similarly, we demonstrated that BTHS CMs showed immature cardiac phenotypes, such as reduced expression of cTnI and MLC2v, as well as abnormal structures unconnected across branches. Thus, BTHS CMs are highly useful for understanding cardiac development and disease modeling in BTHS.

Cardiolipin deficiency induces changes in mitochondrial shape, leading to compromised OXPHOS coupling, reduced mitochondrial membrane potential and increased ROS generation in the mitochondria^20,42^. Consistently, we demonstrated that BTHS CMs exhibited an abnormal mitochondrial structure with a fragmented or swollen morphology, reduced OXPHOS and high levels of ROS. BTHS CMs exhibit reduced levels of mtDNA and mitochondrial markers (MIC60 and TOM20). Cardiolipin deficiency induces alterations in mitochondrial function and an increase in mitochondrial mass^45^. Mitochondrial dysfunction-induced ATP depletion activates AMPK^46^, which directly phosphorylated and activated PGC-1α, the key regulator of mitochondrial biogenesis^47^. Consistently, in the present study, we found that PGC-1α expression is upregulated in BTHS CMs. Despite the increased expression of PGC-1α, the reduced levels of mitochondrial mass in BTHS CMs indicates insufficient mitochondrial biogenesis in the BTHS CMs. Taken together, these results suggest that BTHS CMs are good models for investigating BTHS-associated mitochondrial dysfunction and for developing treatment methods for BTHS.

Injection of viable and respiration-competent mitochondria isolated from normal cardiac tissue into the ischemic zone just before reperfusion significantly enhanced post-ischemic functional recovery and cellular viability in rabbits^48^. Autologous mitochondrial transplantation into the myocardium immediately before reperfusion restores mitochondrial dysfunction in the ischemic heart of pediatric patients^32^. Moreover, the intracoronary delivery of autologous mitochondria after temporary regional ischemia significantly improved myocardial infarction, perfusion and infarct size^49^. However, for clinical applications, the qualities of the isolated mitochondria, including mitochondrial size, membrane potential and morphology, must be carefully monitored, especially if they also contain damaged mitochondria or intracellular organelles such as lysosomes, which may affect the bioenergetic efficiency of the recipient cell or cause cell death. Stem cells have attracted considerable attention as donor cells for allogeneic mitochondrial transplantation^50^. Stem cells have been investigated for their potential to treat ischemia–reperfusion through mitochondrial transfer^51,52^. The present study demonstrated that transplantation of mitochondria isolated from normal CMs mitigated cardiac dysfunction and improved mitochondrial respiratory function in BTHS CMs. iPSC-CMs can be produced from iPS cell clones and possess greater numbers of mitochondria than mesenchymal stem cells. Thus, iPSC-CMs are a good source of mitochondria for allogeneic transplantation.

Mitochondrial transplantation protects against sepsis-induced myocardial dysfunction by modulating mitochondrial biogenesis and fission–fusion of mitochondria^53^. Furthermore, mitochondrial transplantation improved mitochondrial respiratory function by increasing MFN1 expression through the SIRT-1–PGC-1α network^53^. TAZ plays a key role in the execution of mitophagy^23^. Cardiolipin externalization to the outer mitochondrial membrane acts as an ‘eat me signal’ for mitophagy targeting dysfunctional mitochondria to the autophagosomal machinery^54^, which is consistent with the present study suggesting impaired mitophagy in BTHS CMs. Failure to maintain a critical balance between mitophagy and mitochondrial biogenesis leads to the accumulation of dysfunctional mitochondria. In this study, we demonstrated that mitochondrial transplantation induces mitophagy in BTHS CMs, resulting in improved mitochondrial quality and increased expression of mitochondrial proteins. Restoring mitophagy by treatment with rapamycin, an mTORC1 inhibitor, alleviated mitochondrial dysfunction and dilated cardiomyopathy^22^. Furthermore, mitochondrial transplantation upregulated transcriptomic pathways associated with mitochondrial biogenesis^55^. Although the molecular mechanisms underlying mitochondrial transplantation-induced improvement of cardiac and mitochondrial functions are not fully understood, these results suggest a pivotal role of mitophagy and mitochondrial biogenesis in the functional improvement of mitochondria in BTHS CMs. However, we found that mitochondrial transplantation transiently improved mitochondrial functions in BTHS CMs for only 1 week and the transplanted mitochondria were no longer detectable 2 weeks after mitochondrial transplantation. Because BTHS is an X-linked recessive disorder caused by mutations in the *TAZ* gene located on a chromosome^1,56^, the therapeutic effect of mitochondrial transplantation may be limited and gene therapy for the TAZ gene mutation may be necessary to fully restore mitochondrial function in BTHS CMs. The therapeutic effects of allogeneic mitochondrial transplantation on genetic disorders caused by alterations in mitochondrial DNA such as MELAS syndrome (Mitochondrial Encephalopmyopathy, Lactic Acidosis, and Stroke-like episodes) should be further explored to validate the clinical applicability of allogenic mitochondrial transplantation.

In the present study, we demonstrated that allogeneic transplantation of mitochondria isolated from normal CMs ameliorated the mitochondrial and cardiac dysfunction associated with BTHS by stimulating mitophagy. Allogeneic mitochondrial transplantation using iPSC-CMs can be applied not only to BTHS but also to other mitochondrial-dysfunction-related diseases.

## Supplementary information

Supplementary Information

## Data Availability

The Supplementary Information accompanies the manuscript on the *Experimental & Molecular Medicine* website (http://www.nature.com/emm/).

## Study approval

This study was approved by the IRB Boards of Pusan National University (#PNU IRB/2021_25_BR) and Pusan National University Yangsan Hospital (#L-2021-34).

## References

[1] Barth, P. G. et al. An X-linked mitochondrial disease affecting cardiac muscle, skeletal muscle and neutrophil leucocytes. *J. Neurol. Sci.* **62**, 327–355 (1983). 6142097 10.1016/0022-510x(83)90209-5

[2] Neustein, H. B., Lurie, P. R., Dahms, B. & Takahashi, M. An X-linked recessive cardiomyopathy with abnormal mitochondria. *Pediatrics* **64**, 24–29 (1979). 572031

[3] Ferreira, C. et al. *Barth Syndrome* (eds M.P. Adam et al.) (University of Washington, 1993). 25299040

[4] Jarvis, M., Garrett, P. & Svien, L. Gross motor development of a toddler with barth syndrome, an X-linked recessive disorder: a case report. *Pediatr. Phys. Ther.* **13**, 175–181 (2001). 17053636

[5] Vanderniet, J. A., Benitez-Aguirre, P. Z., Broderick, C. R., Kelley, R. I. & Balasubramaniam, S. Barth syndrome with severe dilated cardiomyopathy and growth hormone resistance: a case report. *J. Pediatr. Endocrinol. Metab.* **34**, 951–955 (2021). 33851526 10.1515/jpem-2020-0666

[6] Ronghe, M. D., Foot, A. B., Martin, R., Ashworth, M. & Steward, C. G. Non-Epstein–Barr virus-associated T-cell lymphoma following cardiac transplantation for Barth syndrome. *Acta Paediatr.* **90**, 584–586 (2001). 11430723

[7] Valianpour, F. et al. Linoleic acid supplementation of Barth syndrome fibroblasts restores cardiolipin levels: implications for treatment. *J. Lipid Res.* **44**, 560–566 (2003). 12562862 10.1194/jlr.M200217-JLR200

[8] Rugolotto, S. et al. Long-term treatment of Barth syndrome with pantothenic acid: a retrospective study. *Mol. Genet. Metab.* **80**, 408–411 (2003). 14654353 10.1016/j.ymgme.2003.07.002

[9] Taylor, C. et al. Clinical presentation and natural history of Barth syndrome: an overview. *J. Inherit. Metab. Dis.* **45**, 7–16 (2022). 34355402 10.1002/jimd.12422

[10] Xu, Y., Malhotra, A., Ren, M. & Schlame, M. The enzymatic function of tafazzin. *J. Biol. Chem.* **281**, 39217–39224 (2006). 17082194 10.1074/jbc.M606100200

[11] Vreken, P. et al. Defective remodeling of cardiolipin and phosphatidylglycerol in Barth syndrome. *Biochem. Biophys. Res. Commun.* **279**, 378–382 (2000). 11118295 10.1006/bbrc.2000.3952

[12] Petit, P. X., Ardilla-Osorio, H., Penalvia, L. & Rainey, N. E. Tafazzin mutation affecting cardiolipin leads to increased mitochondrial superoxide anions and mitophagy inhibition in Barth syndrome. *Cells* **9**, 2333 (2020). 33096711 10.3390/cells9102333 PMC7589545

[13] Goncalves, R. L. S., Schlame, M., Bartelt, A., Brand, M. D. & Hotamisligil, G. S. Cardiolipin deficiency in Barth syndrome is not associated with increased superoxide/H_2_O_2_ production in heart and skeletal muscle mitochondria. *FEBS Lett.* **595**, 415–432 (2021). 33112430 10.1002/1873-3468.13973 PMC7894513

[14] Youle, R. J. & Narendra, D. P. Mechanisms of mitophagy. *Nat. Rev. Mol. Cell Biol.* **12**, 9–14 (2011). 21179058 10.1038/nrm3028 PMC4780047

[15] Picca, A., Faitg, J., Auwerx, J., Ferrucci, L. & D’Amico, D. Mitophagy in human health, ageing and disease. *Nat. Metab.* **5**, 2047–2061 (2023). 38036770 10.1038/s42255-023-00930-8 PMC12159423

[16] Youle, R. J. & van der Bliek, A. M. Mitochondrial fission, fusion, and stress. *Science* **337**, 1062–1065 (2012). 22936770 10.1126/science.1219855 PMC4762028

[17] Chu, C. T. et al. Cardiolipin externalization to the outer mitochondrial membrane acts as an elimination signal for mitophagy in neuronal cells. *Nat. Cell Biol.* **15**, 1197–1205 (2013). 24036476 10.1038/ncb2837 PMC3806088

[18] Ghosh, S., Iadarola, D. M., Ball, W. B. & Gohil, V. M. Mitochondrial dysfunctions in barth syndrome. *IUBMB Life* **71**, 791–801 (2019). 30746873 10.1002/iub.2018 PMC6586490

[19] Kiebish, M. A. et al. Dysfunctional cardiac mitochondrial bioenergetic, lipidomic, and signaling in a murine model of Barth syndrome. *J. Lipid Res.* **54**, 1312–1325 (2013). 23410936 10.1194/jlr.M034728 PMC3622326

[20] McKenzie, M., Lazarou, M., Thorburn, D. R. & Ryan, M. T. Mitochondrial respiratory chain supercomplexes are destabilized in Barth syndrome patients. *J. Mol. Biol.* **361**, 462–469 (2006). 16857210 10.1016/j.jmb.2006.06.057

[21] Wang, G. et al. Modeling the mitochondrial cardiomyopathy of Barth syndrome with induced pluripotent stem cell and heart-on-chip technologies. *Nat. Med.* **20**, 616–623 (2014). 24813252 10.1038/nm.3545 PMC4172922

[22] Zhang, J., Liu, X., Nie, J. & Shi, Y. Restoration of mitophagy ameliorates cardiomyopathy in Barth syndrome. *Autophagy* **18**, 2134–2149 (2022). 34985382 10.1080/15548627.2021.2020979 PMC9466615

[23] Hsu, P. et al. Cardiolipin remodeling by TAZ/tafazzin is selectively required for the initiation of mitophagy. *Autophagy* **11**, 643–652 (2015). 25919711 10.1080/15548627.2015.1023984 PMC4502692

[24] Johnson, J. M. et al. Targeted overexpression of catalase to mitochondria does not prevent cardioskeletal myopathy in Barth syndrome. *J. Mol. Cell Cardiol.* **121**, 94–102 (2018). 30008435 10.1016/j.yjmcc.2018.07.001 PMC6178222

[25] Sabbah, H. N. Barth syndrome cardiomyopathy: targeting the mitochondria with elamipretide. *Heart Fail. Rev.* **26**, 237–253 (2021). 33001359 10.1007/s10741-020-10031-3 PMC7895793

[26] McCully, J. D., Levitsky, S., Del Nido, P. J. & Cowan, D. B. Mitochondrial transplantation for therapeutic use. *Clin. Transl. Med.* **5**, 16 (2016). 27130633 10.1186/s40169-016-0095-4 PMC4851669

[27] Norat, P. et al. Mitochondrial dysfunction in neurological disorders: exploring mitochondrial transplantation. *NPJ Regen. Med.* **5**, 22 (2020). 33298971 10.1038/s41536-020-00107-x PMC7683736

[28] Noh, S. E., Lee, S. J., Lee, T. G., Park, K. S. & Kim, J. H. Inhibition of cellular senescence hallmarks by mitochondrial transplantation in senescence-induced ARPE-19 cells. *Neurobiol. Aging* **121**, 157–165 (2023). 36442417 10.1016/j.neurobiolaging.2022.11.003

[29] Sun, C. et al. Endocytosis-mediated mitochondrial transplantation: transferring normal human astrocytic mitochondria into glioma cells rescues aerobic respiration and enhances radiosensitivity. *Theranostics* **9**, 3595–3607 (2019). 31281500 10.7150/thno.33100 PMC6587163

[30] Gollihue, J. L. et al. Optimization of mitochondrial isolation techniques for intraspinal transplantation procedures. *J. Neurosci. Methods* **287**, 1–12 (2017). 28554833 10.1016/j.jneumeth.2017.05.023 PMC5533517

[31] Chang, C. Y., Liang, M. Z. & Chen, L. Current progress of mitochondrial transplantation that promotes neuronal regeneration. *Transl. Neurodegener.* **8**, 17 (2019). 31210929 10.1186/s40035-019-0158-8 PMC6567446

[32] Zhang, Z. et al. Muscle-derived autologous mitochondrial transplantation: a novel strategy for treating cerebral ischemic injury. *Behav. Brain Res.* **356**, 322–331 (2019). 30213662 10.1016/j.bbr.2018.09.005

[33] Shi, Q. et al. Pterostilbene alleviates liver ischemia/reperfusion injury via PINK1-mediated mitophagy. *J. Pharm. Sci.* **148**, 19–30 (2022). 10.1016/j.jphs.2021.09.005 34924126

[34] Wu, Z. et al. Prompt graft cooling enhances cardioprotection during heart transplantation procedures through the regulation of mitophagy. *Cells* **10**, 2912 (2021). 34831135 10.3390/cells10112912 PMC8616468

[35] Jiang, T. et al. Adiponectin ameliorates lung ischemia–reperfusion injury through SIRT1–PINK1 signaling-mediated mitophagy in type 2 diabetic rats. *Respir. Res.* **22**, 258 (2021). 34602075 10.1186/s12931-021-01855-0 PMC8489101

[36] Zhang, H. et al. The role of mitochondria in liver ischemia-reperfusion injury: from aspects of mitochondrial oxidative stress, mitochondrial fission, mitochondrial membrane permeable transport pore formation, mitophagy, and mitochondria-related protective measures. *Oxid. Med. Cell Longev.* **2021**, 6670579 (2021). 34285766 10.1155/2021/6670579 PMC8275408

[37] Kubat, G. B. et al. The effects of mesenchymal stem cell mitochondrial transplantation on doxorubicin-mediated nephrotoxicity in rats. *J. Biochem. Mol. Toxicol.* **35**, e22612 (2021). 32870571 10.1002/jbt.22612

[38] Mori, D. et al. Mitochondrial transfer induced by adipose-derived mesenchymal stem cell transplantation improves cardiac function in rat models of ischemic cardiomyopathy. *Cell Transpl.* **32**, 9636897221148457 (2023). 10.1177/09636897221148457 PMC9834779 36624995

[39] Doenst, T., Nguyen, T. D. & Abel, E. D. Cardiac metabolism in heart failure: implications beyond ATP production. *Circ. Res.* **113**, 709–724 (2013). 23989714 10.1161/CIRCRESAHA.113.300376 PMC3896379

[40] Lian, X. et al. Robust cardiomyocyte differentiation from human pluripotent stem cells via temporal modulation of canonical Wnt signaling. *Proc. Natl Acad. Sci. USA* **109**, E1848–E1857 (2012). 22645348 10.1073/pnas.1200250109 PMC3390875

[41] Kim, Y. S. et al. Tomatidine-stimulated maturation of human embryonic stem cell-derived cardiomyocytes for modeling mitochondrial dysfunction. *Exp. Mol. Med* **54**, 493–502 (2022). 35379934 10.1038/s12276-022-00746-8 PMC9076832

[42] Dudek, J. et al. Cardiolipin deficiency affects respiratory chain function and organization in an induced pluripotent stem cell model of Barth syndrome. *Stem Cell Res.* **11**, 806–819 (2013). 23792436 10.1016/j.scr.2013.05.005

[43] Gonzalez, A., Hall, M. N., Lin, S. C. & Hardie, D. G. AMPK and TOR: the Yin and Yang of cellular nutrient sensing and growth control. *Cell Metab.* **31**, 472–492 (2020). 32130880 10.1016/j.cmet.2020.01.015

[44] Ronvelia, D., Greenwood, J., Platt, J., Hakim, S. & Zaragoza, M. V. Intrafamilial variability for novel TAZ gene mutation: Barth syndrome with dilated cardiomyopathy and heart failure in an infant and left ventricular noncompaction in his great-uncle. *Mol. Genet. Metab.* **107**, 428–432 (2012). 23031367 10.1016/j.ymgme.2012.09.013 PMC3483384

[45] Gonzalvez, F. et al. Barth syndrome: cellular compensation of mitochondrial dysfunction and apoptosis inhibition due to changes in cardiolipin remodeling linked to tafazzin (TAZ) gene mutation. *Biochim. Biophys. Acta* **1832**, 1194–1206 (2013). 23523468 10.1016/j.bbadis.2013.03.005

[46] Hardie, D. G. AMPK: a key regulator of energy balance in the single cell and the whole organism. *Int. J. Obes.* **32**, S7–S12 (2008). 10.1038/ijo.2008.116 18719601

[47] Jager, S., Handschin, C., St-Pierre, J. & Spiegelman, B. M. AMP-activated protein kinase (AMPK) action in skeletal muscle via direct phosphorylation of PGC-1α. *Proc. Natl Acad. Sci. USA* **104**, 12017–12022 (2007). 17609368 10.1073/pnas.0705070104 PMC1924552

[48] McCully, J. D. et al. Injection of isolated mitochondria during early reperfusion for cardioprotection. *Am. J. Physiol. Heart Circ. Physiol.* **296**, H94–H105 (2009). 18978192 10.1152/ajpheart.00567.2008 PMC2637784

[49] Shin, B. et al. A novel biological strategy for myocardial protection by intracoronary delivery of mitochondria: safety and efficacy. *JACC Basic Transl. Sci.* **4**, 871–888 (2019). 31909298 10.1016/j.jacbts.2019.08.007 PMC6938990

[50] Gomzikova, M. O., James, V. & Rizvanov, A. A. Mitochondria donation by mesenchymal stem cells: current understanding and mitochondria transplantation strategies. *Front. Cell Dev. Biol.* **9**, 653322 (2021). 33898449 10.3389/fcell.2021.653322 PMC8058353

[51] Han, H. et al. Bone marrow-derived mesenchymal stem cells rescue injured H9c2 cells via transferring intact mitochondria through tunneling nanotubes in an in vitro simulated ischemia/reperfusion model. *Mol. Med. Rep.* **13**, 1517–1524 (2016). 26718099 10.3892/mmr.2015.4726 PMC4732861

[52] Liu, K. et al. Mesenchymal stem cells rescue injured endothelial cells in an in vitro ischemia-reperfusion model via tunneling nanotube like structure-mediated mitochondrial transfer. *Microvasc. Res.* **92**, 10–18 (2014). 24486322 10.1016/j.mvr.2014.01.008

[53] Mokhtari, B., Hamidi, M., Badalzadeh, R. & Mahmoodpoor, A. Mitochondrial transplantation protects against sepsis-induced myocardial dysfunction by modulating mitochondrial biogenesis and fission/fusion and inflammatory response. *Mol. Biol. Rep.* **50**, 2147–2158 (2023). 36565415 10.1007/s11033-022-08115-4

[54] Chu, C. T., Bayir, H. & Kagan, V. E. LC3 binds externalized cardiolipin on injured mitochondria to signal mitophagy in neurons: implications for Parkinson disease. *Autophagy* **10**, 376–378 (2014). 24351649 10.4161/auto.27191 PMC5396091

[55] Rossi, A. et al. Mitochondria transplantation mitigates damage in an in vitro model of renal tubular injury and in an ex vivo model of DCD renal transplantation. *Ann. Surg.* **278**, e1313–e1326 (2023). 37450698 10.1097/SLA.0000000000006005 PMC10631499

[56] Bione, S. et al. A novel X-linked gene, G4.5. is responsible for Barth syndrome. *Nat. Genet.* **12**, 385–389 (1996). 8630491 10.1038/ng0496-385

