# Supplementary material for: Allogenic mitochondria transfer improves cardiac function in iPS-cell-differentiated cardiomyocytes of a patient with Barth syndrome
Source: Exp Mol Med. 2025 Jun 24;57(6):1260–71. doi: 10.1038/s12276-025-01472-7 (PMC12229508; doi:10.1038/s12276-025-01472-7)
Supplement: Supplementary file 1 — Supplementary Information [file 12276_2025_1472_MOESM1_ESM.pdf]

Supplementary information

Allogenic mitochondria transfer improves cardiac function in iPSC-differentiated cardiomyocytes of a patient with Barth syndrome

Ye Seul Kim[1*], Sukdong Yoo [2*], Yoon Ji Jung[1], Jung Won Yoon[1], Yong Seong Kwon[1], Nayeon Lee[1], Chong Kun Cheon[2,3#], Jae Ho Kim[1#]

[1]Department of Physiology, School of Medicine, Pusan National University, Yangsan, Republic of Korea; [2]Division of Medical Genetics and Metabolism, Department of Pediatrics, School of Medicine, Pusan National University, Pusan National University Children's Hospital, Yangsan, Republic of Korea; [3]Research Institute for Convergence of Biomedical Science and Technology, Pusan National University Yangsan Hospital, Yangsan, Republic of Korea

[*]Equally contributed authors

Supplementary Table 1. Information of Antibodies

Antibodies used for western blot/ immunocytochemistry/ flow cytometry			
Antibody	Dilution		Company Catalog #
	WB	ICC/FC	
cTnT alexa 647		1:1000	PMG-565744
cTnI alexa 488		1:1000	Ab196384
OCT4	1:1000	1:200	Ab181557
SOX2	1:1000	1:200	Ab97959
Nanog	1:1000	1:200	Ab109250
Lin28A		1:200	24017-1-AP
SSEA4		1:200	Sc-21704
GAPDH	1:1000	1:200	SC-47724
cTnT	1:1000	1:200	Ab45932
cTnI	1:1000	1:200	Ab47003
aSA	1:1000	1:200	A7732
MLC2a	1:500	1:200	311011
MLC2v	1:500	1:200	10906-1-AP
TAZ(BTHS)	1:1000		A12722
Mic60	1:1000	1:200	Ab110329
COX4	1:2000		4850S
TTC11/FIS1	1:1000	1:200	Ab96764
MFN1	1:500	1:200	Sc166644
TOM20	1:500	1:100	Sc177764
MFN2	1:1000		Ab56889
DRP1	1:1000		8570S
p-DRP1(Ser616)	1:1000		3455S
OPA1	1:1000		Ab42364
Lamp1	1:1000	1:200	A16894
Phospho-mTOR(Ser2448)	1:1000		5536

mTOR	1:1000		2983
Phospho p70S6K(Thr389)	1:1000		9234
P70S6K	1:1000		9202
SQSTM1/p62	1:1000		AB56416
LC3A/B	1:1000		12741S
Beclin-1	1:1000		3495S
ATG5	1:1000		12994S
VDAC	1:1000		4866
Beta tubulin	1:1000		801201
OXPHOS	1:500		Ab110411
Anti-rabbit IgG H&L(HRP)	1:5000		Ab6802
Anti-mouse IgG H&L(HRP)	1:5000		A17168
ALEXA Fluor 488 Goat anti-mouse		1:1000	A11029
ALEXA Fluor 647 Goat anti-rabbit		1:1000	A21245

Supplementary Table 2. Information of Primers

Primers used for qRT-PCR/ gene mutation analysis		
**Primer**	**Forward**	**Reverse**
TAZ_qRT	AGGAGGGAGCATAGAAGGCA	TCCCTGAGGAGGTAGTGAGC
TAZ_exon	TGAACCACCTGACCGTGCAC	AGAGATGAGGGTCGTCCATG
TAZ_seq	TCTGCACGTGAAGTGGCCGT	GTAGCTCCTTGGTGAAGCAG
PHB2	ACGGCCCTGA AGCTGTTGCT	GAAGTGAAGGCCCTCGGCCA
BCL2L13	TCTGACATTG TGCACGTGGA GA	GTGGCAGTGATATGTGGAAGCA
FUNDC1	AGGTGCTGCT TTGTTCGCTC	TGGCTGGTGT GCAGGATTTC
BNIP3L	ACACGTACCATCCTCATCC TCCA	ATCTTCTTGTGGCGAAGGGCTGT
BNIP3	TGGGTAGAACTGCAC TTCAGCA	TCTTG TGGTG TCTGC GAGCG A
PRKN1	AGCCC CGTCC TGGTT TTCCA	AGGCAGGGAGTAGCCAAGTTGA
PGC1a	ACT GAG CTA CCC TTG GGA TG	TAA GGA TTT CGG TGG TGA CA
GAPDH	GGTGAAGGTCGGAGTCAACGGA	GAGGGATCTCGCTCCTGGAAGA
HK2	TTCAC TTCTT GGTCC CTTTC CA	ACTCA CCCCT CTCCT CTGGA T
UCP2	GTGTGATGGG CACCA TTCTG AC	TGTTTGACAGAATCATACAGGCC
COX2	AGTAC ACCGA CTACG GCGGA C	TCGGG AGTAC TACTC GATTG TC
16srRNA	ACCTA ACAAA CCCAC AGGTC C	CTTAG CATGT ACTGC TCGGA G

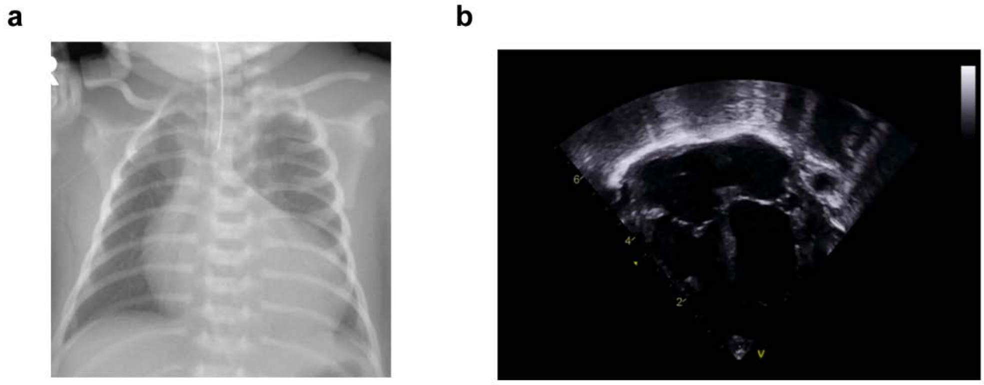

Supplementary Fig. 1. Chest X-ray and Echocardiography Findings.
(a) Chest radiograph performed around 2nd day of birth showing cardiomegaly. (b) Echocardiography revealed an enlarged left ventricle in a patient with BTHS.

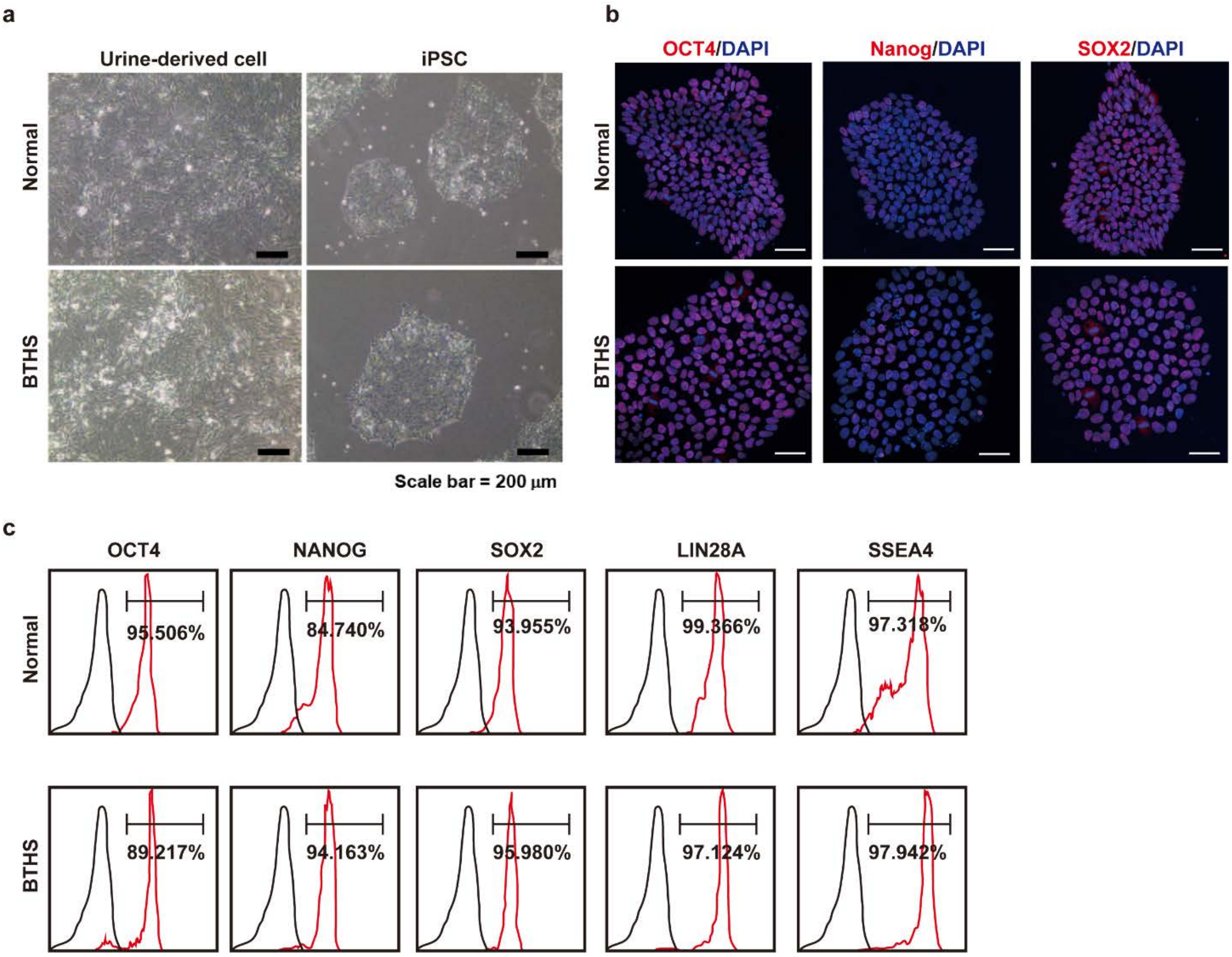

Supplementary Fig. 2. Characteristics of induced pluripotent stem cells.
(a) Images of urine-derived primary cells and iPSCs using a bright-field microscope. Primary somatic cells were isolated from urine of normal and BTHS patient and induced to iPSCs as described in Materials and Methods. The representative images of urine-derived cells and iPSC colonies were photographed. (b) Immunocytochemical analysis of pluripotency markers (OCT4, NANOG, and SOX2) in normal and BTHS iPSCs. The iPSC colonies were stained with antibodies against OCT4, NANOG, and SOX2 (red color) and nuclei were counter-stained with DAPI. The scale bar is 200 μm. (c) Flow cytometric analysis of single cells dissociated from normal and BTHS iPSC colonies. The expression levels of pluripotency markers in normal and BTHS iPSCs were measured by FACS analysis and the percentages of cells expressing pluripotency markers were indicated.

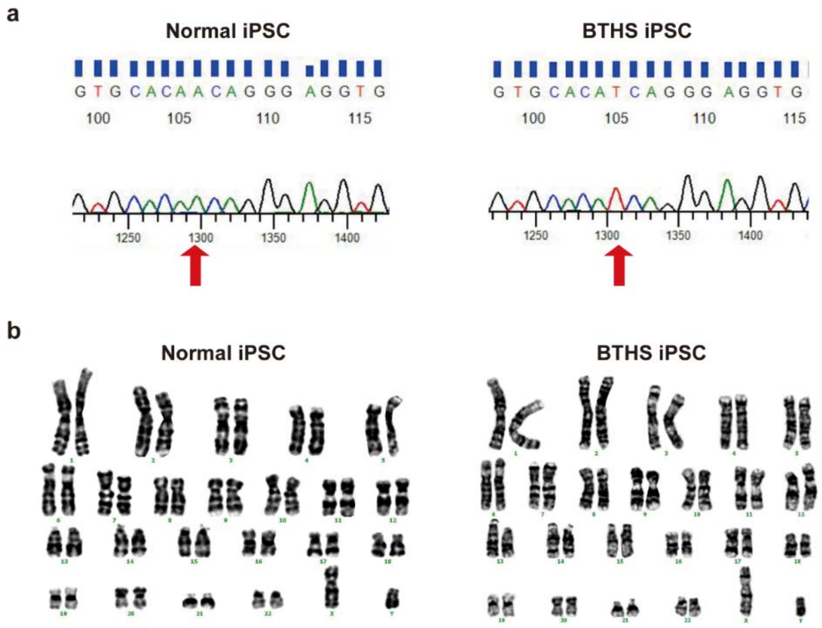

Supplementary Fig. 3. Genetic analysis of normal and BTHS-iPSC. (a) Sequence analysis of the *TAFAZZIN* gene located on Xq28. iPSCs produced from a normal patient and a patient with BTHS and genomic sequences of the *TAFAZZIN* gene located on Xq28 were analyzed at passage 10. Point mutations are indicated in BTHS iPSC with red arrow. (b) Karyotyping of iPSCs showing 46 XY chromosomes.

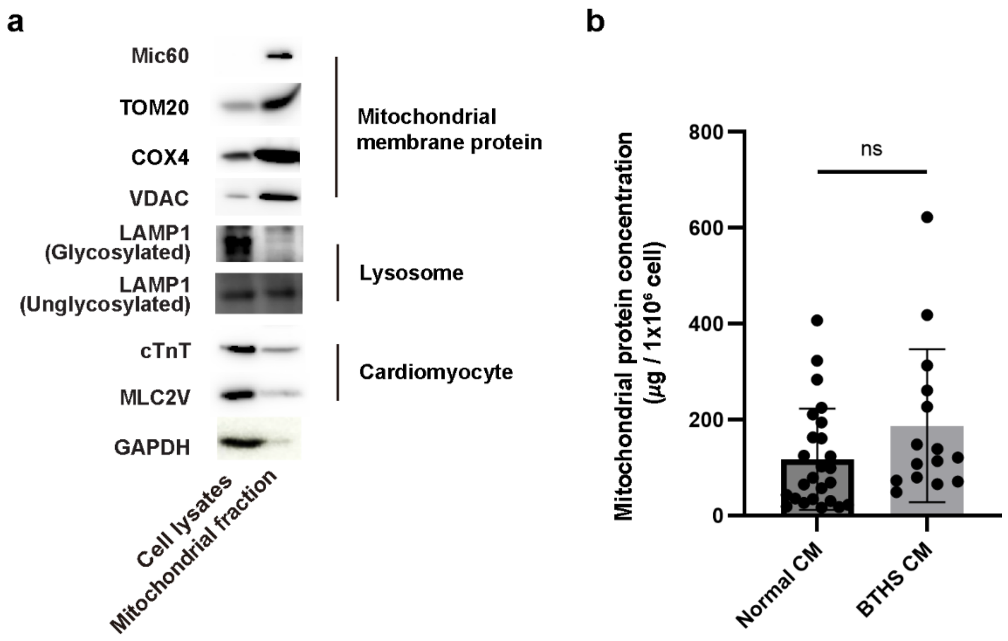

Supplementary Fig. 4. Expression of cardiac and mitochondrial markers in cell lysates and mitochondrial fractions isolated from iPSC-CMs. (a) The protein levels of mitochondrial (Mic60, TOM20, COX4, VDAC), cardiomyocyte (cTnT and MLC2v), and lysosome (LAMP1) were determined in cell lysates and mitochondrial fractions isolated from normal CMs. Representative images are shown from three independent experiments. (b) The protein concentrations of mitochondrial fractions isolated from normal and BTHS CMs were measured using the Bradford assay. Data are presented as mean ± S.D. (n=15~25). Statistical significance was determined using unpaired *t* test with two-tailed P value.

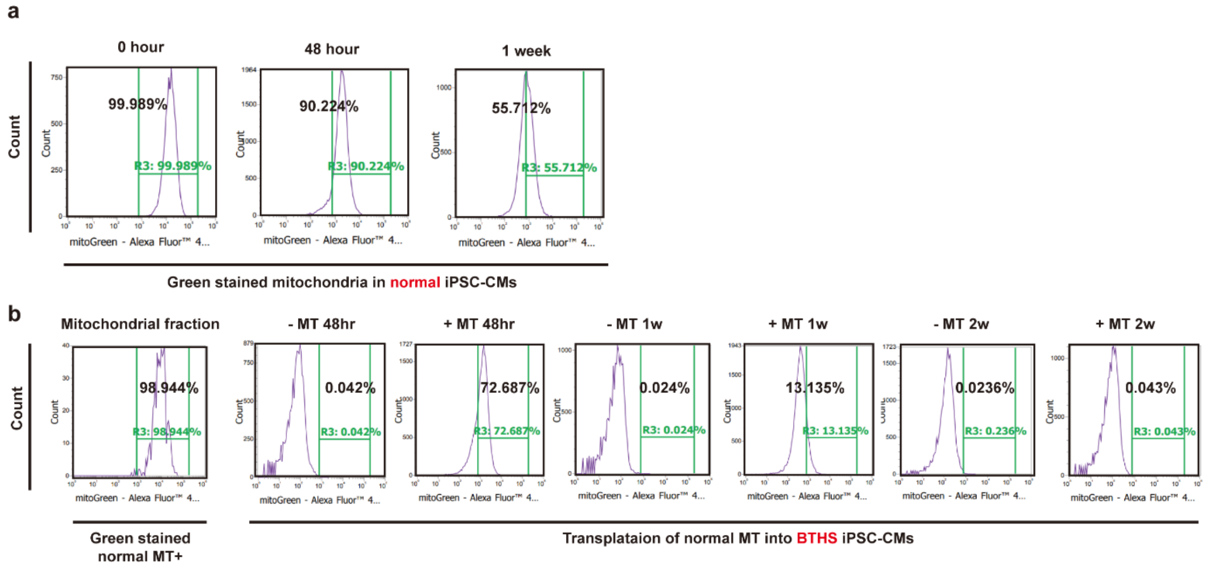

Supplementary Fig. 5. Kinetics of mitochondrial transplantation in BTHS CMs. (a) Time-dependent retention of mitochondrial staining in MitoFlamma Green-stained mitochondria in normal CMs. (b) Treatment time dependence of mitochondrial transplantation efficiency. Mitochondria in normal CMs were labeled with MitoFlamma Green, isolated, and treated to BTHS CMs. The levels of donor mitochondria transplanted into BTHS CMs were determined by FACS analysis at 48 h,1-week, and 2-weeks after mock treatment (-MT) or mitochondrial transplantation (+MT), and the percentages of MitoFlamma Green-positive BTHS CMs were indicated.

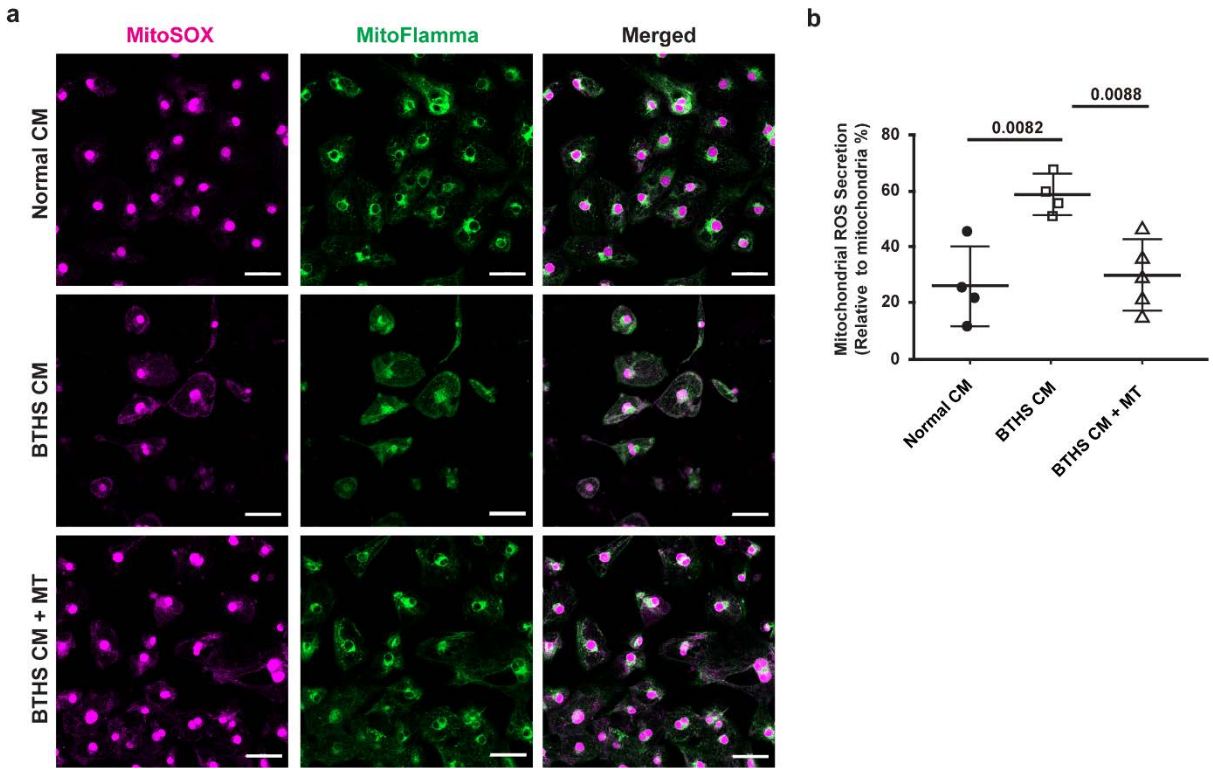

Supplementary Fig. 6. Effect of mitochondrial transplantation on mitochondrial ROS levels in BTHS CMs. (a) Staining of cardiomyocytes with MitoSOX and MitoFlamma Green dyes. Mitochondrial ROS (MitoSOX) were double-stained with mitochondria (MitoFlamma Green) in normal iPSC-CMs (Normal CM), mock-treated BTHS iPSC-CMs (BTHS CM), and mitochondria-transplanted BTHS iPSC-CMs (BTHS CM+MT) and analyzed by confocal microscopy. The scale bar = 20 µm. (b) Quantification of mitochondrial ROS in BTHS CM transplanted with mitochondria. The MitoSOX-positive fluorescence was quantified using ImageJ. Data are presented as mean ± S.D. (n = 4~5). Statistical significance was calculated using by one way-ANOVA with Holm-Sidak's multiple comparisons test and *p* values are shown in the figure.

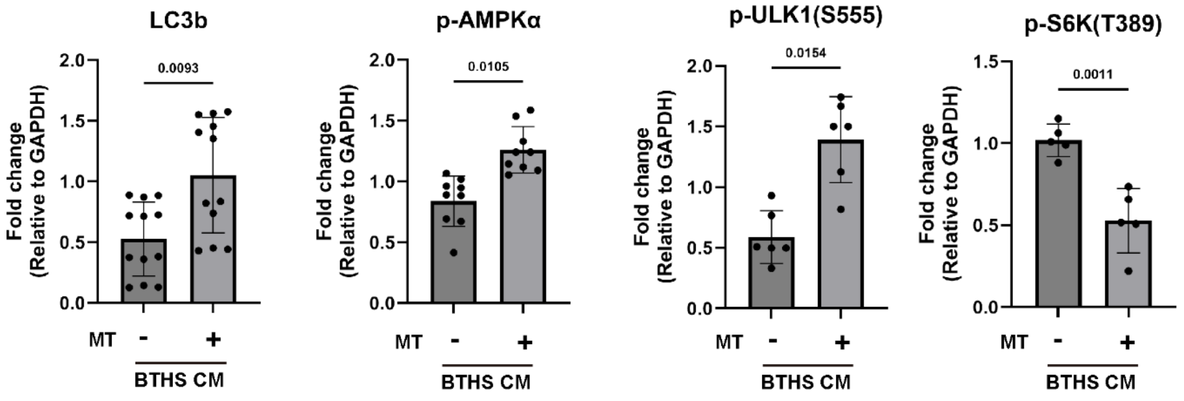

Supplementary Fig. 7. Effects of mitochondrial transplantation on autophagy-related signaling in BTHS CMs. Protein bands of Figure 5c (LC3b, p-AMPKα, p-ULK1, and p-S6K) were quantified using ImageJ software. Data are presented as mean ± S.D (n=6~12). Statistical significance was calculated using by one way-ANOVA with Holm-Sidak's multiple comparisons test and *p* values are indicated in the figure.

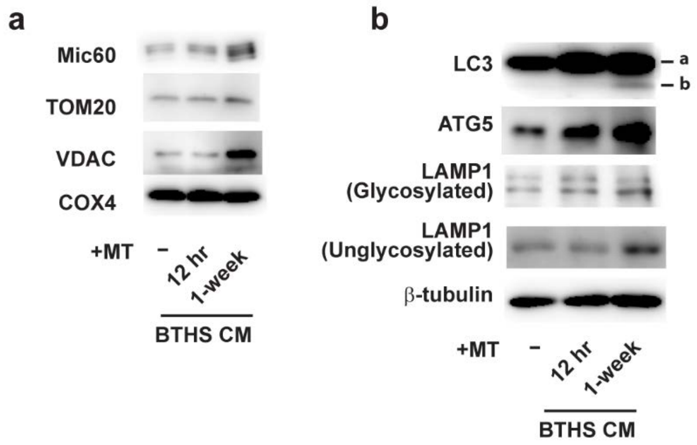

Supplementary Fig. 8. Time-dependent expression of mitophagy-related proteins in BTHS CMs after mitochondrial transplantation. (a) Immunoblotting analysis of mitochondrial membrane proteins in BTHS iPSC-CMs transplanted with normal mitochondria at 12 hours and 1 week after transplantation. (b) Immunoblotting analysis of lysosomal proteins (LC3, ATG5, glycosylated LAMP1, and unglycosylated LAMP1) and β-tubulin in BTHS iPSC-CMs transplanted with normal mitochondria at 12 hours and 1 week after transplantation. The protein bands of LC3a and LC3b were indicated as a and b, respectively.

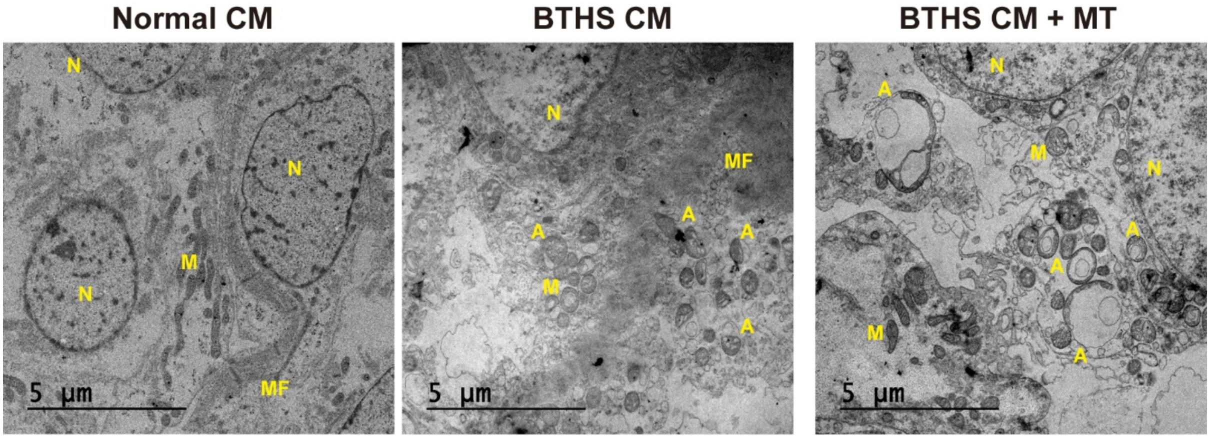

Supplementary Fig. 9. Low-magnification TEM images of mitochondria in mitochondria-transplanted BTHS CM. TEM images of normal CM, BTHS CM, and BTHS CMs 48 h after mitochondrial transplantation (BTHS CM+MT). Scale bar = 5 μm. Abbreviations in images indicate autophagosome-like structure (A), nucleus (N), mitochondria (M), and myofibril (MF).
